# Role of the Sn-TiO_2_/Ti-SnO_2_ Heterojunction in Enhancing the Photocatalytic Oxidation of Arsenite (As^III^) through the Promotion of Charge Carrier Lifetime

**DOI:** 10.1021/acsami.4c14247

**Published:** 2024-12-04

**Authors:** Hany Fathy Heiba, Jay C. Bullen, Andreas Kafizas, Camille Petit, Daqian Jiang, Dominik J. Weiss

**Affiliations:** † Department of Earth Science & Engineering, Imperial College London, London SW7 2AZ, U.K.; ‡ Department of Chemistry, Molecular Science Research Hub, Imperial College London, London W12 0BZ, U.K.; § National Institute of Oceanography and Fisheries, NIOF, Cairo 11516, Egypt; ∥ Department of Civil, Construction, and Environmental Engineering, The University of Alabama, Tuscaloosa, Alabama 35487, United States; ⊥ Grantham Institute, Imperial College London, London SW7 2AZ, U.K.; # Barrer Centre, Department of Chemical Engineering, Imperial College London, London SW7 2AZ, U.K.

**Keywords:** photocatalytic oxidation, charge carrier kinetics, As^III^ remediation, quantum efficiency (QE), heterojunction photocatalyst, reaction mechanism, radical scavenger studies

## Abstract

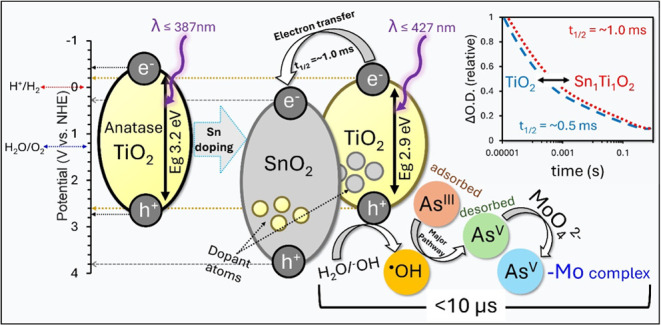

This study proposes the heterojunction photocatalyst, Sn-doped TiO_2_/Ti-doped SnO_2_ (herein named Sn_1_Ti_1_O_2_), as a promising alternative to pure TiO_2_. Sn_1_Ti_1_O_2_ demonstrates improved light harvesting efficiency over TiO_2_ by generating longer-lived electron–hole (e_CB_^–^―h_VB_^+^) pairs, while also displaying a smaller band gap compared to pure TiO_2_. Consequently, we show that it is a promising candidate for the photocatalytic oxidation (PCO) of As^III^ to the less toxic and more readily removable form As^V^. Transient absorption spectroscopy (TAS) shows increased e_CB_^–^―h_VB_^+^ recombination half-lives from ∼0.5 ms in TiO_2_ to ∼1 ms in Sn_1_Ti_1_O_2_. The initial transient absorption signal for Sn_1_Ti_1_O_2_ is twice that of pure TiO_2_, suggesting early time scale (pre-μs) suppression of (e_CB_^–^―h_VB_^+^) recombination. Moreover, TAS showed that Sn_1_Ti_1_O_2_ possesses more reactive charge carriers than TiO_2_ under reactions with chemical scavengers. For the first time, TAS experiments were conducted using both a colorimetric indicator (molybdate) and As^III^ to determine the PCO kinetics from As^III^ to As^V^. The TAS molybdate—As^III^ experiment results indicate that the oxidation process occurs on the sub-microsecond time scale, with a notable increase in absorption at ∼700 nm, providing evidence of the formation of the As^V^—molybdate blue complex. PCO experiments showed that ^•^OH radicals played the predominant role during PCO, followed by superoxide radicals (O_2_^•—^). ^•^OH scavengers including isopropanol, rebamipide anhydrous, and dimethyl sulfoxide (DMSO) reduce the PCO yield of As^III^ to 21, 30, and 23%, respectively. While O_2_^•—^ scavengers including superoxide dismutase (SOD) and *p*-benzoquinone suppressed the PCO yield of As^III^ to a lesser degree, with yields of 35 and 49% seen, respectively. The effects of irradiance intensity, salinity, pH, As^III^ concentration, and photocatalyst mass on both the quantum efficiency (QE) and PCO kinetics were investigated. The Sn_1_Ti_1_O_2_ catalyst exhibited effective recyclability, validating its economical reusability. Overall, the study demonstrates the potential of the Sn_1_Ti_1_O_2_ heterojunction photocatalyst for the PCO of As^III^ to the less toxic As^V^ in water treatment, showing faster oxidation kinetics and improved charge separation compared to pure TiO_2_ as proven by TAS.

## 1 Introduction

Hundreds of millions of people worldwide are exposed to drinking water contaminated with carcinogenic arsenic at concentrations >10 μg/L, the provisional permissible level set by the World Health Organization (WHO).^1^ Inorganic As^III^ is more toxic than As^V^, and is neutrally charged at pH 7, making it difficult to remove using conventional water treatment technologies such as precipitation, coagulation, adsorption, and ion exchange, unlike negatively charged As^V^ oxyanions. Oxidation of the more prevalent As^III^ to less toxic As^V^ is therefore crucial to enable effective water treatment.^2^ Heterogeneous photocatalytic oxidation (PCO) provides a sustainable and eco-friendly solution,^3^ without the risk of generating toxic disinfection byproducts (DBPs) associated with chemical homogeneous oxidants. However, TiO_2_-based PCO of As^III^ remains a challenging process due to the band gap limitation (3.2 eV), where the most studied anatase form can only harvest ∼2% of sunlight, and exhibits relatively short-lived charge carriers (e_CB_^–^―h_VB_^+^) that results in poor quantum efficiency (QE).^3,4^

Our previous study demonstrated that the heterojunction between Fe_2_O_3_ and TiO_2_ enhances overall arsenic removal via adsorption. However, the PCO showed a substantial decrease (up to 60-fold) due to the parasitic light absorption by the Fe_2_O_3_ component, with 88% of photons being absorbed at 368 nm alongside fast (e_CB_^–^―h_VB_^+^) recombination in this phase.^5^ Here, we seek to address the challenge by establishing a heterojunction that prevents charge carrier recombination,^6^ where a promising Sn-doped-TiO_2_/Ti-doped-SnO_2_ (Sn_1_Ti_1_O_2_) heterojunction is explored in detail.^3^ The choice of Sn and Ti as dopants for SnO_2_ and TiO_2_, respectively, was based on their ability to form a thermodynamically favorable type II heterojunction structure when combined. Specifically, Sn doping of TiO_2_ and Ti doping of SnO_2_ result in a staggered band alignment that promotes spatial separation of photogenerated charge carriers (e_CB_^–^―h_VB_^+^).^7,8^ This increases charge carrier lifetime and the probability of generating more reactive oxygen species (ROS)^5,9^ which ultimately enhances PCO efficiency.

Our previous measurements of the energy levels in TiO_2_ and SnO_2_ show that such a heterojunction would form a type II structure,^3^ where electrons transfer from the lower work function TiO_2_ (ϕ = 4.2 eV) to the higher work function SnO_2_ (ϕ = 4.9 eV).^10^ Therefore, SnO_2_ acts as a sink for photogenerated electrons from TiO_2_, while TiO_2_ acts as a sink for photogenerated holes from SnO_2_.^3^ Such a TiO_2_–SnO_2_ heterojunction possesses a sufficient oxidative potential to oxidize water, where the valence band (VB) maximum of both TiO_2_ and SnO_2_ are more positive than the potential required to oxidize water and generate hydroxyl radicals (^•^OH/OH^–^ at +2.59 V vs NHE) and the conduction band (CB) minimum of TiO_2_ is more negative than the potential needed to reduce oxygen and form superoxide radicals (O_2_/O_2_^•—^ at −0.16 V vs NHE).^11^

Previous studies have demonstrated TiO_2_–SnO_2_ to show superior photocatalytic activity than naked TiO_2_ due to the enhancement of charge carrier separation in the heterojunction.^12^ However, TiO_2_–SnO_2_ is promising for gas sensing,^10^ solar cells,^13,14^ batteries,^15,16^ and water treatment (e.g., organic dyes,^17^ pharmaceuticals,^18^ herbicide^19^), it has not been studied for the remediation of AsIII contaminated water and charge carrier lifetimes has not been studied yet.

To guide the rational design of advanced photocatalyst materials for the PCO of As^III^, the study of charge carrier dynamics is crucial.^20^ Interestingly, this aspect has not been studied in this reaction. Furthermore, to our knowledge, both the PCO of As^III^ and the charge carrier dynamics of the TiO_2_–SnO_2_ heterojunction system has not previously been studied. In addition, although the PCO of As^III^ has extensively been studied for TiO_2_, the identification of the oxidant responsible for the rate-limiting step (i.e., As^III^ → As^IV^) remains a controversial issue. There is no consensus on the exact identity of the major oxidant species, whether it is ^•^OH, HO_2_^•^, and O_2_^•—^ radicals or directly via h_VB_^+^.^21,22^ Furthermore, As^V^ is well known to selectively bind to molybdate solution and form the As^V^-molybdate blue complex, which absorbs light in the near-red region. The formation of this complex provides evidence of As^III^ oxidation to As^V^ and could be exploited. However, the real-time measurement of the oxidation kinetics of this reaction, specifically upon the production of As^V^ in the presence of the catalyst, As^III^ and molybdate reagent, remains unexplored.

This study aims to address the aforementioned knowledge gaps. First, transient absorption spectroscopy (TAS), (a widely recognized pump–probe spectroscopy technique used to track the dynamics of charge carriers, including their generation, migration, trapping, and recombination, in metal oxide semiconductor photocatalysts^23−25^), is applied to monitor the charge carrier dynamics during the PCO of As^III^ over the Sn_1_Ti_1_O_2_ heterojunction photocatalyst and the PCO kinetics of As^III^. To the best of our knowledge, this is the first study to examine the charge carrier dynamics of this heterojunction system and the *in situ* measurements of PCO kinetics of As^III^ to As^V^ over time scales relevant to catalysis, ranging from microseconds to seconds. Second, the identification of the main oxidant species during the oxidation process has been investigated using different kinds of radical scavengers. Third, the influence of reaction controls (e.g., irradiance power, salinity, pH, photocatalyst mass to solution ratio, and As^III^ concentration) on both the quantum efficiency (QE) and PCO of As^III^ is systematically studied. By achieving these goals, this research aims to aid in the development of more efficient arsenic treatment facilities for water purification systems.

## 2 Materials and Methods

### 2.1 Synthesis of TiO_2_, SnO_2_, and Sn_1_Ti_1_O_2_

#### 2.1.1 Sn_1_Ti_1_O_2_ and TiO_2_

A Sn-doped TiO_2_/Ti-doped SnO_2_ heterojunction, with a 1Sn:1Ti ratio was synthesized using sol–gel synthesis method^26^ where tin tetrachloride (SnCl_4_) [98%, Sigma-Aldrich] and titanium(IV) *tert*-butoxide (Ti[OC(CH_3_)_3_]_4_) [>98%, Sigma-Aldrich] precursors. This heterojunction is herein referred to as Sn_1_Ti_1_O_2_. A 3 M solution of SnCl_4_ (3.91 g) in deionized water (5.0 mL) was added dropwise at room temperature to a 1 M solution of Ti[OC(CH_3_)_3_]_4_ (5.12 mL) in ethanol (15.0 mL) [>99.9%, VWR]. After continuous stirring for 2 h, a sol was produced then left for 24 h at room temperature with stirring. The obtained xerogel was subsequently placed in a crucible and calcined in the furnace at 450 °C for 4 h. The calcined powder was ground using an agate mortar to obtain a fine powder.^26^ A pure TiO_2_ reference was prepared by substituting the 3 M SnCl_4_ solution in water for deionized water (5.0 mL).

#### 2.1.2 SnO_2_

A pure SnO_2_ reference sample was synthesized following sol–gel methods in the literature.^27−29^ A transparent sol solution was prepared by dissolving 5 g of SnCl_4_ in 100 mL of absolute ethanol under vigorous magnetic stirring for 30 min. At a controlled rate, ammonia solution [25%, Sigma-Aldrich] was then added dropwise while stirring until reaching a pH of ∼8–9. Afterward, the hydrolysis and condensation reactions were initiated by adding 10 mL of deionized water dropwise under continuous stirring for 2 h. The resulting gel was then dried at 70 °C for 1 h to remove traces of ethanol impurities. The powder was then calcined at 400 °C for 2 h in air. The resulting material is referred to as SnO_2_.

The details of the purity, grades, and suppliers of the chemicals and reagents used in this study are listed in Table S1.

### 2.2 Material Characterization

TiO_2_, SnO_2_, and Sn_1_Ti_1_O_2_ photocatalysts were characterized using energy dispersive X-ray spectroscopy (EDX), X-ray photoelectron spectroscopy (XPS), low energy ion scattering (LEIS), X-ray diffraction (XRD), scanning electron microscopy (SEM) and high-resolution transmission electron microscopy (HR-TEM). A conclusion of the characterization findings is summarized in the Supporting Information (Section 2. Summary of Characterization findings) while the full characterization details can be found in our recently published work.^3^

### 2.3 Transient Absorption Spectroscopy (TAS)

The experimental setup used herein, was as reported by Jiamprasertboon et al.,^30^ however some alterations were made: (i) the frequency of the 355 nm laser pulse was increased to ∼1 Hz, (ii) the measurements of the diffuse reflectance were between 600 and 1000 nm using 100 nm increments, and (iii) 100 laser pulses were used to calculate each kinetic trace, except for experiments in As^III^ suspensions. Here, to reduce the effect of As^III^ photooxidation due to the laser pulse, 10 pulses were collected at each wavelength (in a randomized order), with triplicate measurements (a total of 30 pulses at each wavelength). Samples were measured as (a) a dry film in air, (b) in water, (c) in 2 mM AgNO_3(aq)_, (d) in methanol, and (e) 1 mg/L As^III^ in molybdenum blue solution.

The optical density change (ΔOD) at time (*t*), and wavelength (λ), is related to the change in the intensity of light (transmitted or reflected), through the eq 1:
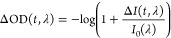
1where *I*_0_ is the intensity of light (transmitted or reflected) before the laser pulse.^31^

Treating SnO_2_, TiO_2_, and Sn_1_Ti_1_O_2_ phases within the composite photocatalyst as noninteracting components, a component additive prediction was calculated to assess the prevalence of charge transfer across the Sn_1_Ti_1_O_2_ heterojunction.^5^ Where charge transfer across the heterojunction occurs, charge carrier lifetimes will be longer lived than charge carriers generated in the single-component reference samples, and consequently the experimentally observed TAS decay will be slower (intense signals lasting until longer times) than in the TAS decay predicted by the component additive sum of the single-component reference TAS decays.^5^ First, to determine the half-lives of the transient absorption, we modeled the transient absorption decay curves using a power-law decay, shown in eq 2.
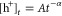
2where [h^+^]*_t_* is the concentration of positive holes at time *t* (defined by the change in optical density, ΔOD), *t* is time (seconds), and *A* & α are the fitting parameters.^32^ Parameters *A* & α were optimized to improve the (*R*^2^) fit between experimental and modeled data. For these fittings, data before 0.1 and after 100 ms were excluded due to interference from the laser pulse and vibrational noise, respectively. TAS spectra were standardized to unity at *t* = 100 μs with the normalized value of *A* calculated via eq 3:
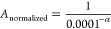
3The half-lives (*t*_1/2_) of TAS spectra were finally determined by reordering eqs 2 to 4.
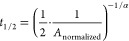
4

### 2.4 Photocatalytic Oxidation and Adsorption of As^III^

A stock solution of 1000 mg/L As^III^ was prepared by dissolving 0.66 g of As_2_O_3_ [99.99%, trace metal basis, Sigma-Aldrich] in a beaker containing 5 mL of 1 M NaOH [>99%, trace metal basis, VWR], followed by shaking for 30 s, then transfer to a 500 mL volumetric flask and complete to the mark with deionized water (Milli-Q). A suspension of 100 mL of 10 mg/L As^III^ was added to 0.01 g of the photocatalyst, and the mixture was stirred magnetically for 2 h. The pH was adjusted to 7.41 using small volumes of 1 M HCl/1 M NaOH. Working solutions of As^III^ were prepared by sequential dilution of the stock solutions. The molybdenum blue method was used to determine the concentration of As^V^ in the solution.^2^ A molybdenum analytical solution (100 mL) was prepared by subsequently dissolving 0.5 g ammonium molybdate [99.98%, trace metal basis, Sigma-Aldrich], 0.04 g potassium antimony tartrate [>99%, AnalaR], 4 mL conc. H_2_SO_4_ [(95–99) %, VWR] and 0.5 g of ascorbic acid [≥99.0%, ACS reagent, Sigma-Aldrich] in deionized water.

Photocatalytic oxidation (PCO) experiments were conducted for the TiO_2_, SnO_2_, and Sn_1_Ti_1_O_2_ photocatalysts under UV light. The distance between the UV lamp (18 W UVA lamp, 370 nm; Sylvania) and the sample surface was 1.8 cm (unless otherwise mentioned). At this distance, the light intensity at the surface of the TiO_2_ suspension was 5.59 mW cm^–2^ (cf. measurement details in Section 2.5). The surface area of the reactor was 20.4 cm^–2^, giving a light intensity of 114 mW over the surface of the reaction. The reaction was performed in duplicate, with UV irradiation being present in one reaction to determine the PCO fraction of As^III^ and no UV irradiation (i.e., dark) in the other reaction to evaluate the adsorption fraction of As^III^.^2^

Suspensions of a specified As^III^ concentration and a specified weight of photocatalyst (in 100 mL total volume) were prepared, with the pH adjusted by using small volumes of 1 M HCl and 1 M NaOH. Suspensions were stirred magnetically. The values of concentrations and photocatalyst weight between (0.02 to 0.4) g/L in addition to other factors are mentioned in each Figure caption. Aliquots (2 mL) were collected at predetermined time intervals (1, 3, 5, 10, 20, 40, 60, and 120 min) and centrifuged to separate the photocatalyst from the aqueous phase. Finally, the supernatant was analyzed for arsenic species.

The effect of the direct adsorption of As^III^ was corrected by running two simultaneous experiments with Sn_1_Ti_1_O_2_ one under UV and the second in the dark. The PCO yield was calculated with a correction for the directly adsorbed As^III^ (*i*.*e*. the decline in aqueous As^III^ that is not due to oxidation). This correction was applied at each time interval throughout the course of the experiment using eq 5.
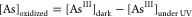
5

The concentration of As^V^ was determined by adding 80 μL of the previously prepared molybdenum analytical solution to 1 mL of each aliquot, shaken for 1 min, and left for 1 h. The absorbance was then measured at 882 nm using a Jenway 7315 UV–vis spectrophotometer. The total arsenic concentration [As(T)] in the solution was determined by oxidizing all of the sample’s As^III^ species to As^V^ by adding 120 μL of potassium permanganate (KMnO_4_) [99%, Sigma-Aldrich] to 1 mL of each aliquot, and then shaking for 1 min. The sample was left for 1 h to allow for complete oxidation, and then the amount of As^V^ (representing total As) was determined using the aforementioned molybdenum blue method.

The PCO rate constant (*k*_1_ min^–1^) was calculated using the first-order kinetic rate law eq 6.
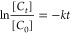
6where *C*_0_ and *C*_t_ represent arsenic concentrations (μg/L) at the initial time (0) and at time (t) respectively. The standard deviation was calculated based on the linear fitness of the points.

During the study of the seawater salinity effect, artificial seawater was prepared according to the American Society for Testing and Materials protocol: ASTM D1141-98.

### 2.5 Radical Scavenging Experimental Setup

Radical scavenging experiments were conducted to identify the main reactive oxidative species involved in the photocatalytic oxidation (PCO) of As^III^ using the Sn_1_Ti_1_O_2_ photocatalyst. A solution containing 20 mg/L As^III^ at pH 7.4 was prepared with 0.01 g/100 mL of Sn_1_Ti_1_O_2_ and irradiated with UVA light (λ_max_ = 365 nm). It is worth noting that we opted for 20 mg/L in the scavenging experiments for two main reasons. First, when using 10 mg/L, we observed that the reaction proceeded too quickly, particularly with electron scavengers, making it challenging to capture data effectively. Second, employing a higher concentration allowed us to better monitor and compare changes across various experiments. Various scavengers were employed to examine their effects on the PCO reaction. Silver nitrate (2 mM AgNO_3_ [≥99.0% (ACS reagent), Sigma-Aldrich]) and ascorbic acid (2 mM, [≥99.0% (ACS reagent), Sigma-Aldrich]) were used to scavenge electrons (e_CB_^–^) and holes (h_VB_^+^), respectively. Rebamipide anhydrous (67.4 μM [≥98%, HPLC grade, Sigma-Aldrich]) and dimethyl sulfoxide (1.4 M DMSO [>99%, HPLC grade, VWR]) served as hydroxyl radical (^•^OH) scavengers. Finally, Superoxide (O_2_^•—^) was scavenged using superoxide dismutase (0.25 mM SOD [Assay Kit, Sigma-Aldrich]) and *p*-benzoquinone (2 mM, [≥98%, reagent grade, Sigma-Aldrich]).

### 2.6 Irradiation Power Measurement

The lamp power was measured by using an optical power meter (PM 100, Thorlabs) connected to a power sensor (S120UV, Thorlabs). To accurately measure the lamp power, visible light reaching the power meter should be excluded. Therefore, its lamp power (mW/cm^2^) is the difference between the lamp power without a light filter and the lamp power while applying a long pass (LP) Filter <400 nm [that allows all light with λ > 400 nm to pass through]. Then the calculated lamp power was divided by the detector area and finally, the output was multiplied by the photocatalysis reactor area. The calculations are provided in eq 7.
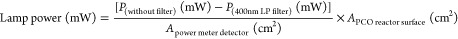
7where *P*_(without filter)_ is the lamp power without using a light filter, *P*_(400 nm LP filter)_ is lamp power while applying 400 nm LP light filter, *A*_(power meter detector)_ is the power meter detector area and *A*_(PCO reactor surface)_ is the surface area of the PCO reactor being exposed to the light. The lamp power was measured at different distances and then converted to the photon flux. For example, at a distance of 1.8 cm between the lamp and the solution surface, *P*_(without applying light filter)_ = 6.201 mW, *P*_(LP Filter 400 nm)_ = 1.811 mW, *A*_(power meter detector area)_ = (0.79 cm^2^) and the PCO reactor surface area = 20.4 cm^2^. Therefore, the light irradiation was 5.59 mW·cm^–2^ and the total irradiance received by the PCO reactor surface was 114.03 mW.

### 2.7 Determination of the Quantum Efficiency of the PCO of As^III^

The spectral output of the UV lamp used in our PCO experiments was measured using an Ocean Optics detector (USB4000-VIS-NIR) equipped with a fiber optic cable (QP600-2-UV/BX) and processed using Spectra Suite software, scanning its emission from 350 to 1000 nm. The light intensity was measured by using an optical power meter (PM 100, Thorlabs) connected to a power sensor (S120UV, Thorlabs).

To determine the photon flux, various short-pass filters were used to prevent visible light of the lamp from reaching the power meter (λ < 400 nm, λ < 475 nm, and λ < 610 nm). Emission spectra were adjusted to achieve the corresponding measured photon energy output (*J*) using eq 8:
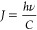
8where *h* represents the Planks constant, ν is the frequency in (Hz), and *c* is the speed of light in cm·s^–1^. Lamp power was then converted to photon flux (cm^–2^·s^–1^) by using eq 9:
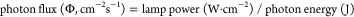
9The optical transmittance of powder suspensions of our photocatalyst (0.01 g/100 mL) was measured using a Shimadzu UV-2700 UV Vis spectrophotometer equipped with an integrating sphere. The transmittance was converted to molar absorptivity (ε, M^–1^ cm^–1^) at different pathlengths (*l*) using the Beer–Lambert law (eq 10):
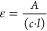
10where *A* is the absorbance, *C* is the molar concentration, and *l* is the path length in cm.

The photon flux (cm^–2^·s^–1^) absorbed at a given depth was determined using eq 11:
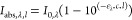
11where *I*_abs,λ_ represents the photon fraction absorbed at the wavelength (λ) and path length (*l*), *I*_o,λ_ is the overall emitted photon flux at λ, ε_λ_ (M^–1^ cm^–1^) is the molar absorption coefficient, *c* (M) is the concentration of TiO_2_ in water, and *l* (cm) is the path length of the light solution.

The quantum yield (Ø) expresses the ratio between the number of converted species and the number of photons absorbed by the photocatalyst (eq 12):
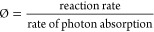
12where the reaction rate and the photon absorption rate are given by eqs 13 and 14, respectively:
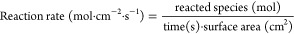
13
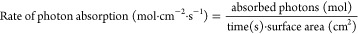
14

## 3 Results and Discussion

### 3.1 Mechanism of the PCO of As^III^

#### 3.1.1 Transient Absorption Spectroscopy (TAS) and Charge Carrier Lifetime

**Figure 1:**
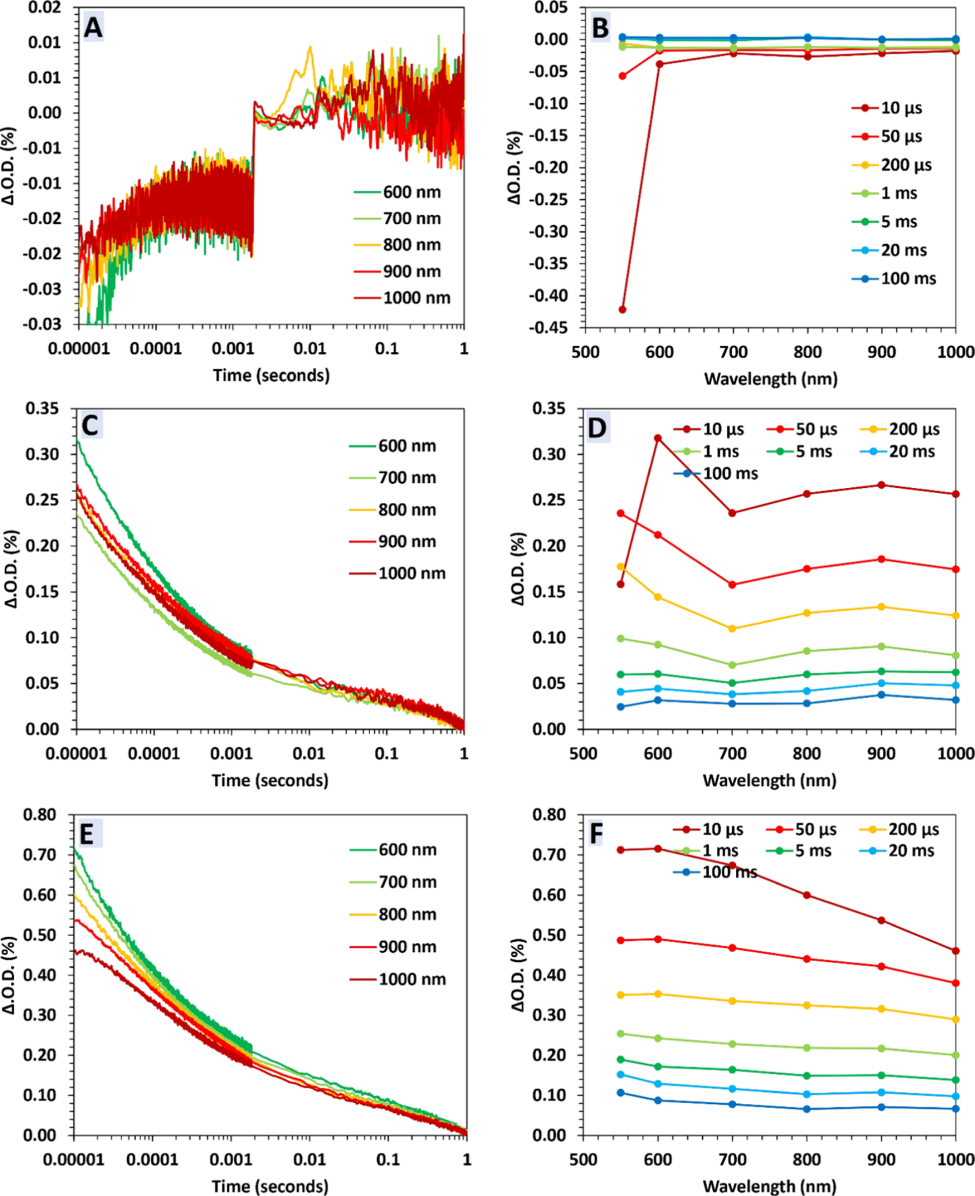
TAS of dry powder showing the changes in absorption vs time for SnO_2_ (A), TiO_2_ (C), and Sn_1_Ti_1_O_2_ (E) and changes in absorption vs wavelength at selected times for SnO_2_ (B), TiO_2_ (D), and Sn_1_Ti_1_O_2_ (F). The operating conditions are as follows: λ_exc_ = 355 nm, ∼1.0 mJ cm^–2^ pulse^–1^, 6 ns pulse width, and laser repetition rate ∼1 Hz. (TAS at 550 nm is presented in Figure S1).

Our recent study found that the band gaps of pure TiO_2_ and SnO_2_ are 3.20 and 3.75 eV, respectively. Sn doping narrowed the band gap from 3.20 eV in TiO_2_ to about 2.90 eV Sn_1_Ti_1_O_2_, which can significantly impact the charge carrier lifetime.^3^ Using TAS, we first aimed to identify any increase in the charge carrier lifetime that may be present in the Sn_1_Ti_1_O_2_ heterojunction. The TAS experiments of dry powder samples of SnO_2_, TiO_2_ and Sn_1_Ti_1_O_2_ are shown in Figure 1. In the absence of any chemical substrates with which the photocatalyst can react, TAS monitors the intrinsic e_CB_^–^―h_VB_^+^ recombination behavior in these materials. For the SnO_2_ sample, almost negligible transient absorption signals are observed, with only slight bleaching observed at 550 nm (*i*.*e*. a negative transient absorption signal, which arises when there is a loss in the ground state absorption). This bleach signal is attributed to shallow intrabandgap defect states in SnO_2_ (*e*.*g*. oxygen vacancy states), which trap photogenerated charge carriers up to the ∼100 μs. For TiO_2_, the typical transient absorption and recombination behavior is observed for this anatase form, i.e., a broad absorption seen across the visible and a recombination half-time of ∼0.5 ms.^24^ For Sn_1_Ti_1_O_2_, a similar transient absorption spectrum and recombination dynamics are observed. However, the initial transient absorption signal of Sn_1_Ti_1_O_2_ is twice that of TiO_2_, which demonstrates that early time scale (pre-μs) e_CB_^–^―h_VB_^+^ recombination is suppressed in the composite system and is evidence of enhanced spatial separation of e_CB_^–^―h_VB_^+^ in this system. Several reasons have been proposed to explain the suppression of e_CB_^–^―h_VB_^+^ recombination, including TiO_2_ passivating recombination sites on SnO_2_, the creation of a redox gradient that forms a barrier preventing electrons in the core (SnO_2_) from reaching oxidized species in the shell, and the trapping of electrons in an electronic state between the core and the shell.^7^ A fact that remains true is the energy offset of the conduction (CB) and valence bands (VB) of SnO_2_ and TiO_2_. This offset forms a type II staggered heterojunction that provides a thermodynamic driving force for charges to spatially separate, with electrons in TiO_2_ “encouraged” to migrate into SnO_2_ and holes in SnO_2_ “encouraged” to migrate into TiO_2_.

The broad TA signals seen in Figure 1 are often a combination of electron and hole carrier signals, where TiO_2_ holes typically absorb in the blue region, and electrons typically absorb in the red region. The regions of the TA spectrum that correspond to electron and hole carriers can be distinguished through the use of hole and electron chemical scavengers, respectively. The most notable electron and hole chemical scavengers, used herein, are silver nitrate and methanol.

It is worth noting that the increase in the absorption value of TiO_2_ from 550 to 600 nm at 10 μs (Figure 1D) is attributed to the fact that 550 nm is approaching the band gap edge of the material. At this point, there is often overlap between bleach states (which represent the loss of ground state excitation) and photoinduced absorption states (where charge carriers that form absorb light in the visible region). This phenomenon is particularly pronounced in early times.^25^ The early time trap state filling and relaxation dynamics are not observed at later times, when the charge carriers have dispersed more uniformly.

TAS experiments of powders suspended in aqueous solutions with AgNO_3_ (the electron scavenger) are presented in Figure S2. It is worth noting that the solution turns darker here over time due to Ag precipitation, which perturbs the system.^25^ Lower signal-to-noise ratios are observed in these experiments due to the tendency of catalyst particles to settle during the measurements. For SnO_2_, no significant changes are observed in the transient decay dynamics, indicating a poor reaction of photogenerated electrons with AgNO_3_. In both our TiO_2_ and Sn_1_Ti_1_O_2_ samples, similar yet marginal electron scavenging behavior is observed, prolonging transient decay signals to the ∼1 ms time scale. Compared to previous studies of more nanoparticulate TiO_2_, the increase in hole carrier lifetime herein is significantly lower, as lifetimes of up to ∼1 s have previously been observed.^25^ Moreover, a minimal change in the transient absorption spectra is observed in these samples, which is indicative of a low quantum efficiency of this scavenging reaction in comparison to that of e_CB_^–^―h_VB_^+^ recombination.

TAS experiments of powder suspensions with methanol, the hole scavenger, are shown in Figure S3. SnO_2_ shows no significant changes in the transient decay dynamics, once again indicating a poor reaction of photogenerated holes with methanol. In TiO_2_, some degree of hole scavenging is observed, with transient decays showing half times of ∼1 ms, which are prolonged until ∼10 ms. In contrast to observations with nanoparticulate TiO_2_, where lifetimes of up to ∼1 s have previously been found,^23^ this increase in electron carrier lifetime is significantly lower. For the Sn_1_Ti_1_O_2_ sample in the presence of the hole scavenger, enhanced transient absorption was seen in red, indicative of the formation of a significant population of electron carriers. Half times were extended from ∼1 ms in air to ∼100 ms in the presence of the hole scavenger.

**Figure 2:**
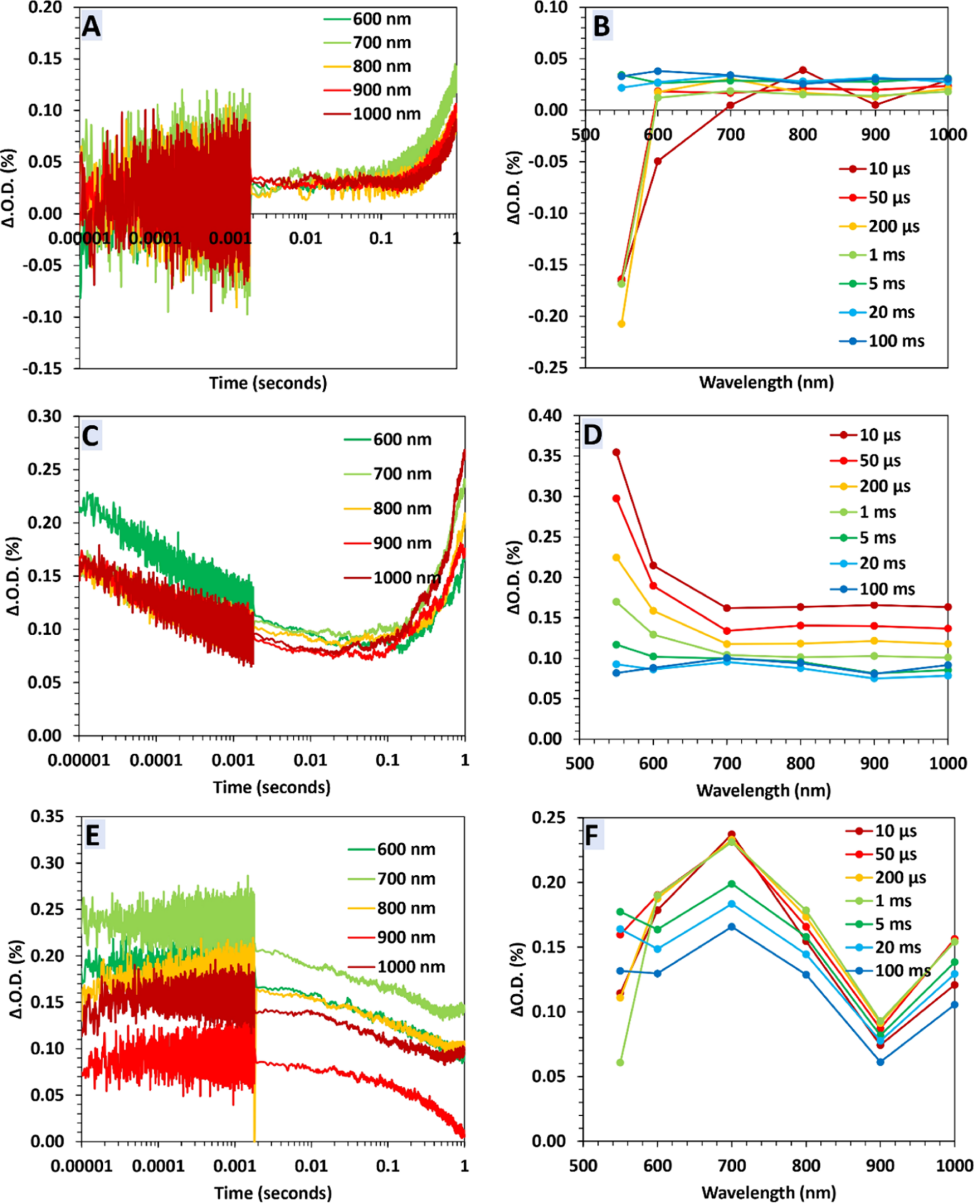
Transient absorption spectroscopy of powder suspensions (1 g/L) in an aqueous solution containing 1 mg/L As^III^ and molybdate complex that forms a blue-colored compound when bound to As^V^. The changes in absorption vs time for SnO_2_ (A), TiO_2_ (C), and Sn_1_Ti_1_O_2_ (E) and changes in absorption vs wavelength at selected times for SnO_2_ (B), TiO_2_ (D), and Sn_1_Ti_1_O_2_ (F). The operating conditions are as follows: λ_exc_ = 355 nm, ∼1.0 mJ cm^–2^ pulse^–1^, 6 ns pulse width, and laser repetition rate ∼1 Hz. (TAS at 550 nm is presented in Figure S4).

TAS experiments of powder suspensions in the presence of 1 mg/L of As^III^ and molybdate reagent are shown in Figure 2. SnO_2_ shows no significant changes in the transient decay dynamics, indicating a poor reaction of photogenerated charges with As^III^ to form As^V^ (*i*.*e*. no observed change in color due to the formation of a blue-colored complex). TiO_2_ shows a similar transient absorption behavior to that in the presence of molybdate but with an increased absorption in the blue region. This indicates a marginal scavenging effect (*i*.*e*. marginal scavenging of charge carriers in TiO_2_ by As^III^). However, the Sn_1_Ti_1_O_2_ sample displays large changes in both transient decay kinetics and transient absorption spectra with half times in the region of ∼100 ms and a stark increase in absorption at ∼700 nm. This is a strong indication that molybdate is reacting with As^V^ in situ during the reaction, giving rise to an increase in transient absorption in the red. Importantly, as this transient absorption peak is observed on the time resolution of this measurement (∼10 μs), this indicates that the oxidation of As^III^ to As^V^ by this photocatalytic material occurs on the pre-μs times, with the subsequent formation of the molybdate complex with As^V^ also being similarly rapid. To the best of our knowledge, this is the first use of a colorimetric indicator to carry out an operando measurement of the kinetics of As^III^ oxidation.

#### 3.1.2 Main Oxidant Species during the PCO

Photocatalytic reactions are complex. The initial step of As^III^ oxidation is the absorption of photons by the heterogeneous photocatalyst to generate oxidant species, such as h_VB_^+^, ^•^OH, and HO_2_^•^/O_2_^•—^ as presented in Table 1 (eqs 15 to 28). Typically, one reaction is rate-limiting and will control the photocatalytic oxidation process (i.e., As^III^ → As^IV^), as presented in (eqs 29 to 32). The reactive intermediate can be determined using scavenger experiments, assessing changes in charge carrier lifetimes and As^III^ oxidation kinetics when different reagents are added to react with, and thereby remove, h_VB_^+^, ^•^OH, or HO_2_^•^/O_2_^•—^ intermediates.

**Table 1:** Charge Carrier Generation and Possible As^III^ Photooxidation Pathways

eqn.	reaction	rate (k)	reference
	*Charge Separation and generation of charge carriers*		
15	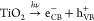 15	*k* = 10^15^ s^–1^	(41)
16	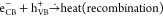 16		(42)
17	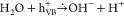 17		(40)
18	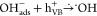 18		(42)
19	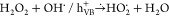 19		(43)
20	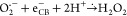 20		(43)
21	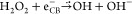 21		(43)
22	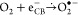 22	*k* = 2.0 × 10^10^ M^1^ s^–1^	(40)
23	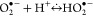 23		
24	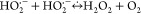 24		
25	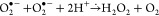 25		
26	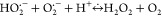 26		
27	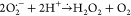 27	*k* = 8.3 × 10^5^ M^–1^ s^–1^	(44)
28	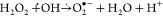 28	*k* = 3 × 10^7^ M^–1^ s^–1^	(45)
*Arsenite oxidation*			
	*Hydroxyl radicals as an oxidant*		
29	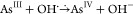 29	*k* = 8.5 × 10^9^ M^–1^ s^–1^	(40)
	*Positive hole as an oxidant*		
30	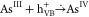 30	*k* = 8.2 × 10^5^ M^–1^ s^–1^	(43,46)
	*Superoxide as an oxidant*		
31	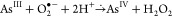 31	*k* = 3.6 × 10^6^ M^–1^ s^–1^	(43)
32	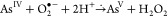 32		
*Other possible oxidation reactions*			
33	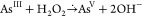 33	*k* = 15.3 M^–1^ s^–1^	(43,46)
34	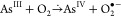 34		
35	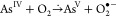 35	*k*_5_ = 1.1 × 10^9^ M^–1^ s^–1^	(43)
*Possible surface processes*			
36	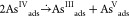 36	*k* = 8.4 × 10^8^ M^–1^ s^–1^	(46)
37	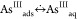 37		(47)
38	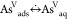 38		(47)
*Possible reduction processes*			
39	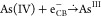 39	*k* = 2.0 × 10^8^ s^–1^	(43)

##### 3.1.2.1 Electron Scavenging

**Figure 3:**
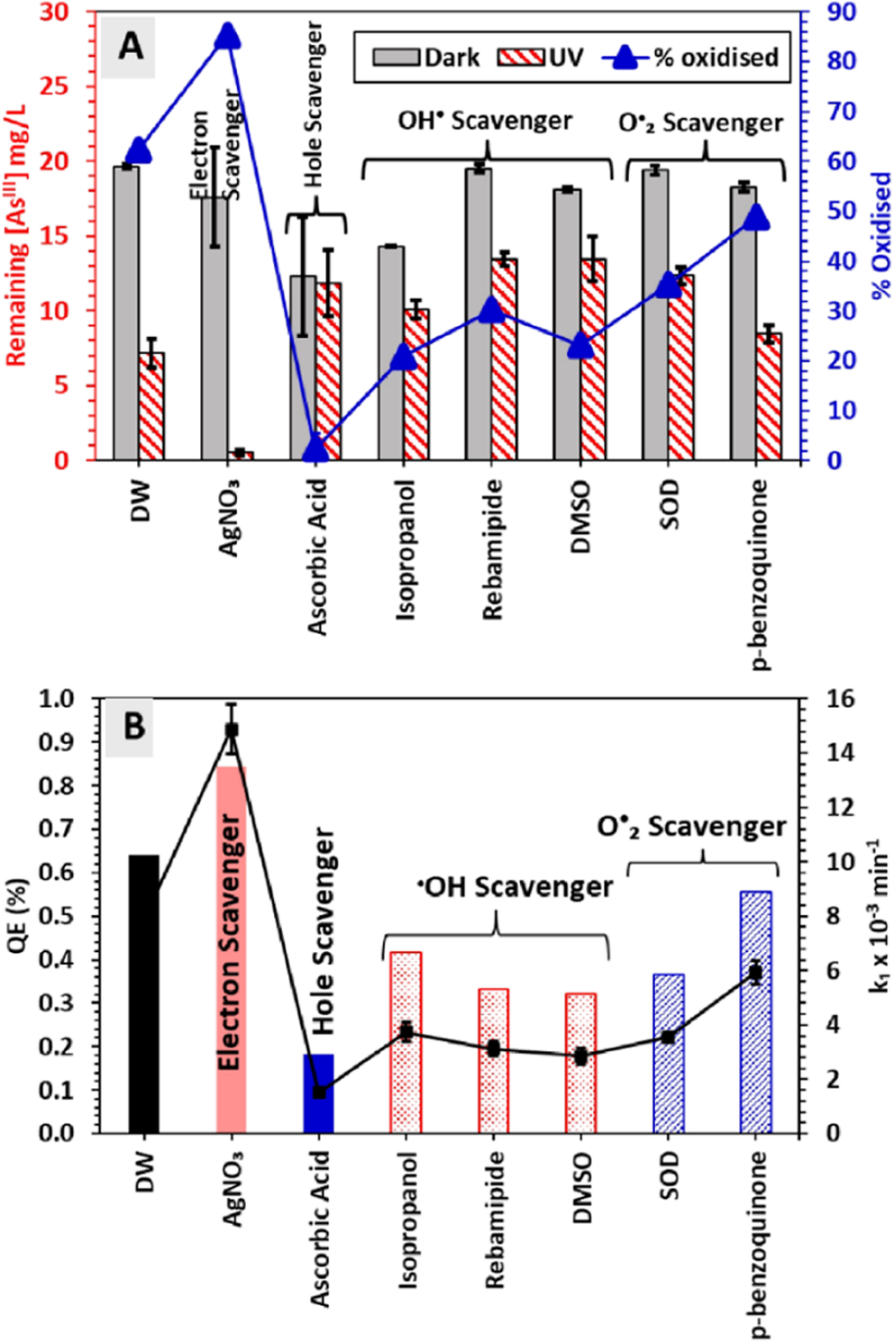
PCO of As^III^ with Sn_1_Ti_1_O_2_ using e_CB_^–^ scavenger (2 mM AgNO_3_), h_VB_^+^ scavenger (2 mM ascorbic acid), ^•^OH scavengers (isopropanol, 67.4 μM rebamipide anhydrous and 1.4 M DMSO), and O_2_^•–^ scavengers (0.25 mM SOD and 2 mM *p*-benzoquinone); (A) Remaining [As^III^] under UV, remaining [As^III^] in the dark and the line is absolute PCO (%), (B) QE (%) and the line is the first order kinetic rate (*k*_1_) plot. [As^III^ = 20 mg/L, 100 mL, pH 7.4, 0.01 g Sn_1_Ti_1_O_2_, *t* = 120 min]. The error bars represent the ± SD values.

As shown in Figure 3 and Table 1, in the control experiment, without a scavenger, the efficiency of the PCO of As^III^ over Sn_1_Ti_1_O_2_ is 62% [*k* = (7.88 ± 0.19) × 10^–3^ min^–1^, QE (%) = 0.64]. Using AgNO_3_ as an electron scavenger increases the oxidation rate to 85.2% [*k* = (14.88 ± 0.89) × 10^–3^ min^–1^, QE (%) = 0.84]. The redox potential of Ag^+^ to Ag is more positive than that of O_2_ to O_2_^•—^, resulting in a stronger thermodynamic driving force for the reaction between e_CB_^—^ and Ag^+^. Additionally, compared to O_2_, the higher concentration of Ag^+^ ions in the solution contributes to the preferential scavenging of e_CB_^–^, leading to a darkening of the solution. Therefore, the enhanced oxidation rate is because AgNO_3_ captures e_CB_^–^, hindering (e_CB_^–^―h_VB_^+^) recombination, increasing the lifetime of h_VB_^+^ and thus allowing for the production of more ^•^OH radicals,^33^ (eq 18). In addition, in Sn_1_Ti_1_O_2_, the CB edge is located at ca. - 0.51 V (at pH 7), which gives the e_CB_^–^ a mildly reducing control. In most PCO processes, molecular oxygen dissolved in water serves as an acceptor of CB electrons (E_1_(O_2_/O_2_^•—^ = −0.33 V). The presence of alternative electron acceptors such as Ag^+^ may accelerate the photocatalytic processes even in the absence of O_2_.^34^ As an electron scavenger, AgNO_3_ also reduces the level of accumulation of photogenerated electrons on the Sn_1_Ti_1_O_2_ surface. Since electrons react preferentially with Ag^+^, leading to the deposition of Ag^0^ solids on the photocatalyst surface, Ag coats the photocatalyst surface and therefore inhibits subsequent production of HO_2_^•^ & O_2_^•—^ (see eqs 21 & 22) and further As^III^ oxidation (see eqs 31 and 32). Although AgNO_3_ will likely inhibit the PCO of As^III^ by the O_2_^•—^ pathway, the overall oxidation increases. This result implies that O_2_^•—^ is not the main oxidant during the PCO of As^III^ over Sn_1_Ti_1_O_2_, while ^•^OH and/or h_VB_^+^ have a more dominant role in the PCO of As^III^.^35^ It is worth noting that AgNO_3_ reacts with arsenic trioxide (As_2_O_3_ [As^III^]) to form stable silver arsenite (Ag_3_AsO_3_ [As^III^]) therefore, it does not affect the oxidation state of As^III^.^36^

##### 3.1.2.2 Hole Scavenging

Ascorbic acid as h_VB_^+^ scavenger dramatically reduces the PCO efficiency to 2.3% [*k* = (1.52 ± 0.14) × 10^–3^ min^–1^, QE (%) = 0.1; Figure 3], suggesting that As^III^ photooxidation is largely driven by h_VB_^+^ and/or its subsequent reactions that form the ^•^OH radical (eq 18).

##### 3.1.2.3 Hydroxyl Scavenging

To explore the contribution of ^•^OH in the oxidation, isopropanol, rebamipide anhydrous, and DMSO were used as ^•^OH scavengers. The PCO efficiency is reduced to 21% [k = (3.74 ± 0.36) × 10^–3^ min^–1^, QE (%) = 0.42] using isopropanol, 30% [*k* = (3.12 ± 0.26) × 10^–3^ min^–1^, QE (%) = 0.33] using rebamipide anhydrous and 23% [*k* = (2.84 ± 0.29) × 10^–3^ min^–1^, QE (%) = 0.32] using DMSO (Figure 3B). It is crucial to emphasize that the employed scavengers do not influence the oxidation states of As^III^/As^V^, given that their redox potentials are not adequately positive to oxidize As^III^ (for example, Isopropanol/acetone at −0.12 V vs SHE;^37^ DMSO/DMSO^–^ at +0.16 V vs SHE;^38^ and As^III^/As^V^ at +0.56 V vs SHE^39^).

##### 3.1.2.4 Superoxide Scavenging

The use of superoxide scavengers, SOD and *p*-benzoquinone (*E*^0^ = −0.198 V < *E*^0^(As^III^/As^V^) = +0.56 V) reduced the oxidation yield to 35.25% [*k* = (3.54 ± 0.17) × 10^–3^ min^–1^, QE (%) = 0.37] and 48.93% [*k* = (5.94 ± 0.43) × 10^–3^ min^–1^, QE (%) = 0.56] respectively. This confirms that ^•^OH scavengers suppress PCO of As^III^ more than O_2_^•—^ scavengers.

The results, summarized in Table S2, conclude that in the studied Sn_1_Ti_1_O_2_ system, ^•^OH is the likely main oxidant followed by direct oxidation by h_VB_^+^. This is supported by the fact that the rate constant for the reaction of ^•^OH and the h_VB_^+^ with As^III^ is ∼3 times faster than its reaction with O_2_^•—^ (see eqs 29 to 31).^40^

**Figure 4:**
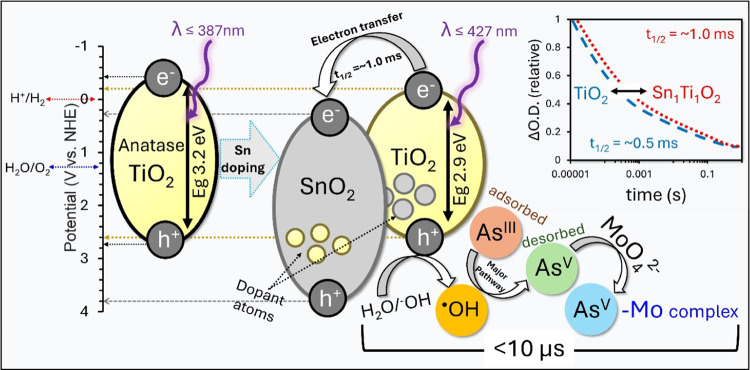
Schematic representation of the PCO mechanism of As^III^ to As^V^ using THE Sn_1_Ti_1_O_2_ heterojunction, emphasizing the roles of charge carrier kinetics, energy band alignment, and ^•^OH as the main oxidant.

Overall, the introduction of Sn dopant into the TiO_2_ lattice significantly influences the photophysical properties of the photocatalyst. The schematic representation displayed in Figure 4 illustrates how Sn doping introduces new electronic energy levels and effectively narrows the band gap from 3.2 eV in TiO_2_ to 2.9 eV in the Sn_1_Ti_1_O_2_ heterojunction. This reduction in band gap leads to a red shift in the absorption edge, changing from λ ≤ 387 nm for TiO_2_ to λ ≤ 427 nm for Sn_1_Ti_1_O_2_ resulting in minimizing the energy requirement for the photoexcitation of electrons from VB to CB while maintaining sufficient oxidative potential to oxidize water and generate ROS. The new band alignment at the Sn_1_Ti_1_O_2_ interface facilitates the efficient transfer of photoexcited electrons from the CB of TiO_2_ to the lower-lying CB of the SnO_2_ domains. This favorable energetic alignment promotes the spatial separation of photogenerated e_CB_^–^―h_VB_^+^ pairs, leading to an extended charge carrier lifetime that increases from approximately 0.5 ms in unmodified TiO_2_ to about 1.0 ms in the Sn_1_Ti_1_O_2_ heterojunction, as evidenced by TAS.

The prolonged charge carrier lifetime in Sn_1_Ti_1_O_2_ enhances the PCO performance, which is further supported by PCO scavenger experiments. Scavenging studies indicate that the ^•^OH radical is the primary oxidizing species responsible for the PCO of As^III^ to As^V^ over the Sn_1_Ti_1_O_2_ photocatalyst. The oxidized As^V^ species can then complex with molybdate (MoO_4_^2–^) to form a distinctive blue arseno-molybdate complex, which enables reliable analytical detection and quantification of the oxidation process.

### 3.2 Control of Experimental Parameters Affecting PCO Efficiency

Initially, baseline control experiments were conducted to evaluate different aspects of the photocatalytic system. The contribution of direct UV photolysis was assessed (Figure S5A), showing a limited extent of As^III^ oxidation (<4.37%) after 2 h. These results suggest that the conversion process proceeds slowly under UV photolysis in the absence of the photocatalyst. The adsorption of As^III^ over Sn_1_Ti_1_O_2_ photocatalyst was studied using an identical setup under dark conditions (Figure S5A) showing negligible As^III^ adsorption (<1%) and low adsorption capacity (Qe = 0.64 mg/g), highlighting the necessity for photocatalysis. Additionally, the photocatalytic performances of Sn_1_Ti_1_O_2_ and its pure oxide forms were also compared (Figure S5B). PCO using Sn_1_Ti_1_O_2_ led to a rapid decrease in As^III^ concentration with ∼83% oxidation achieved within 120 min. In contrast, SnO_2_ performed poorly (∼13%) while TiO_2_ oxidized only 26%. This enhancement associated with Sn_1_Ti_1_O_2_ validates the synergistic effects arising from heterojunction formation. Furthermore, the role of O_2_ during photocatalysis was explored (Figure S5C) by purging the solution with N_2_ for 20 min to remove dissolved O_2_ prior to PCO with Sn_1_Ti_1_O_2_. As^III^ oxidation significantly decreased from ∼83 to 66.7%, demonstrating the dependence of the PCO process on the dissolved O_2_ as a crucial control in driving the photocatalyzed reactions, which reaffirms the previously discussed ROS scavengers’ findings.

#### 3.2.1 Effect of Irradiation Power on PCO Efficiency

**Figure 5:**
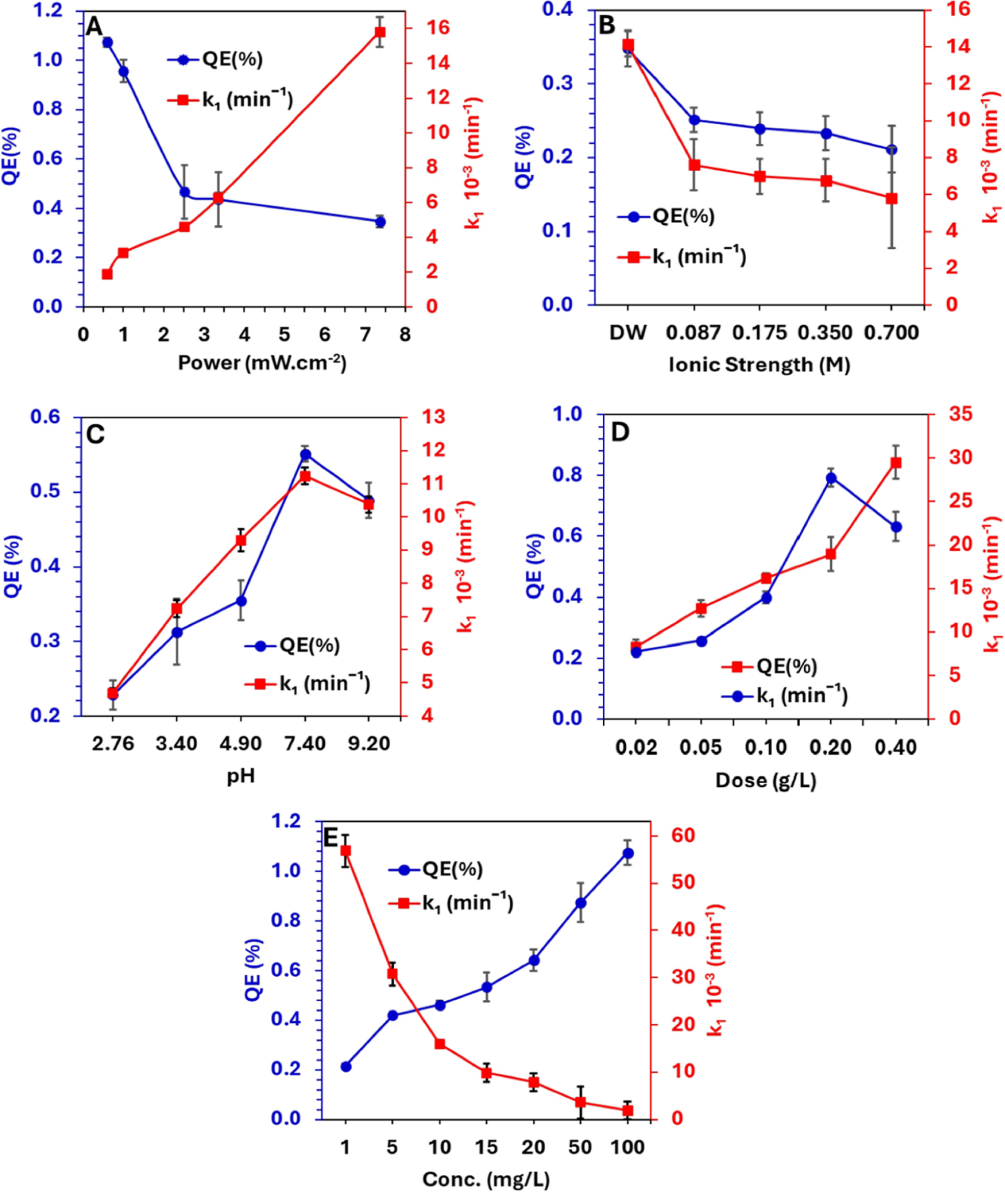
Effect of operating parameters on the kinetic rate constant (*k*_1_) and the quantum efficiency (QE%); (A) Effect of the irradiance power (at 10 mg/L, pH 7.4, 0.1 g/L Sn_1_Ti_1_O_2_), (B) effect of salinity (at 10 mg/L, pH 7.4, 0.1 g/L Sn_1_Ti_1_O_2_, irradiation 7.40 mW/cm^2^), (C) effect of pH (at 10 mg/L, 0.1 g/L Sn_1_Ti_1_O_2_, irradiation 7.40 mW/cm^2^), (D) effect of Sn_1_Ti_1_O_2_ dose (at 10 mg/L, pH 7.4, irradiation 7.40 mW/cm^2^), and (E) effect of [As^III^] concentration (at pH 7.4, 0.1 g/L Sn_1_Ti_1_O_2_, irradiation 7.40 mW/cm^2^). The error bars represent the standard deviation (±SD), and the presented data are the absolute oxidation yield after subtracting the directly adsorbed As^III^ in dark conditions (Figure S11 exhibits the raw data showing the adsorbed [As^III^] in the dark, the decrease in [As^III^] under UV irradiation, and the absolute oxidized [As^III^]).

The light intensity and thus the photon flux arriving at the solution strongly influence the quantum efficiency (QE, %) in heterogeneous photocatalytic oxidation reactions. The range of studied irradiation densities was from 0.59 to 7.40 mW/cm^2^ while reducing the distance between the irradiation source and the solution surface from 22 to 1.8 cm, respectively. As a result, the photon flux (flux·cm^–2^·s^–1^) arriving at the solution surface increased from 1.10 × 10^15^ (at 22 cm) to 1.38 × 10^16^ (at 1.8 cm) as presented in Figure S6. Although the PCO rate constant decreased with light power from (16 ± 0. 85) × 10^–3^ min^–1^ (using 7.4 mW/cm^2^, at 1.8 cm) to (1.9 ± 0.01) × 10^–3^ min^–1^ (using 0.59 mW/cm^2^, at 22 cm,), the QE in fact increased from 0.37 to 1.07%, as shown in Figure 5A. At shorter distances, a higher photon flux arrives at the catalyst surface and generates excess electron–hole (e_CB_^–^―h_VB_^+^) pairs, and given the initial As^III^ concentration is kept constant for this set of experiments, this leads to a high e_CB_^–^―h_VB_^+^ to As^III^ ratio. Therefore, given the lower probability for interactions with As^III^, a greater proportion of e_CB_^–^―h_VB_^+^ pairs recombine without participating in oxidation reactions, resulting in a lower QE.

#### 3.2.2 Effect of Salinity on the PCO Efficiency

The changes in the salinity of groundwater contaminated with As^III^ due to climate change can potentially affect the PCO treatment of groundwater. To study the effect of salinity on the PCO of As^III^ over Sn_1_Ti_1_O_2_, artificial seawater was prepared and diluted to different ionic strength. The results shown in Figure 5B (and Figure S7) indicate that the PCO rates decline from (14 ± 0.7) × 10^–3^ min^–1^ (for the control experiment using distilled water) to (5.8 ± 0.3) × 10^–3^ min^–1^ (*I* = 0.7 M). High salinity suppresses the PCO, where salts likely become adsorbed to the surface and significantly reduce the possibility of As^III^ interaction with the catalyst surface ultimately suppressing PCO efficiency.^48^ The QE decreases with increasing ionic strength for several reasons. Increased ionic strength is recognized for its role in reducing dissolved oxygen levels in solution. This high ionic strength correlates with enhanced ion adsorption on the photocatalyst surface, which significantly lowers the adsorption of dissolved O_2_ on the catalyst,^49^ thereby inhibiting the generation of reactive oxygen species. Furthermore, elevated ionic strength is associated with increased Cl^–^ concentrations, which deactivate h^+^ ions and quench ^•^OH radicals,^50^ ultimately leading to a decrease in quantum efficiency.

#### 3.2.3 Effect of pH on PCO Efficiency

The pH is a crucial parameter during the photocatalytic oxidation (PCO) of As^III^. It can influence the process in two ways. First, the pH controls the protonation and distribution of arsenite species. Second, the pH regulates the availability of hydroxyl ions (^—^OH), which are essential to generate the ^•^OH radicals that drive the overall oxidation process. Careful optimization and management of the pH are necessary to achieve effective As^III^ oxidation through photocatalytic means. The influence of pH was studied and the progress of As^III^ oxidation was 43, 58, 67, 82, and 93%, at pH 2.76, 3.4, 4.9, 7.4, and 9.2 respectively (Figure 5C and Figure S8A,B). The increase in the PCO rate with an increase in pH is possibly due to several factors that control As^III^ to As^V^ redox reactions. The adsorption/desorption process of As^III^ and As^V^ is strongly dependent on the solution pH. In acidic pH, the photocatalyst surface has a stronger affinity toward As^V^ which could saturate the surface and cause photocatalyst fouling^5^ but in slightly alkaline pH, As^III^ is strongly adsorbed and consequently oxidized.^51^ In addition, a higher pH likely promotes the generation of more hydroxide anions (^−^OH) that react with h_VB_^+^ and form ^•^OH which is the main oxidant during the PCO^52^ (Figure 3 and Table S2). It is worth noting that the pH values dropped from 2.8 to 2.9 and from 9.2 to 7.6 after the 2 h PCO experiment (Figure S8C) which is attributed to the consumption of ^•^OH radicals during the oxidation process. The QE increases with pH up to 7.4 but decreases at pH 9.2. At lower pH levels, the conduction and valence band edges of the photocatalyst are shifted to more positive potentials, reducing the driving force for redox reactions.^53^ The decrease in QE at pH 9.2 may be attributed to the reduced stability of the photocatalyst under alkaline conditions, where the isoelectric point of TiO_2_ typically ranges from 4 to approximately 8, while that of SnO_2_ falls between 2 and around 6.^54^

#### 3.2.4 Effect of the Sn_1_Ti_1_O_2_ Concentration on PCO Efficiency

The effect of catalyst loadings ranging between 0.02 and 0.4 g/L on the PCO of 10 mg/L As^III^ is presented in Figure S9. The PCO increases from 40% [*k* = (7.7 ± 0. 14) × 10^–3^ min^–1^ with QE (%)] at 0.02 g/L to 96% [*k=* (27.8 ± 1.1) × 10^–3^ min^–1^ with QE (%)] at 0.2 g/L, and then slightly decreases to 92% [*k* = (22.1 ± 1.7) × 10^–3^ min^–1^ with QE (%)] at 0.4 g/L (Figure 5D). The increase of the PCO rate and QE observed with increasing the photocatalyst loading from 0.02 to 0.2 g/L is explained by (1) a higher photocatalyst concentration allows absorption of a higher proportion of the incident light, which leads to higher rates of e_CB_^–^―h_VB_^+^ generation for redox reactions;^55^ and (2) a higher catalyst concentrations provide more active sites available at the photocatalyst surface, which in turn increases the number of ^•^OH formed.^56^ At extremely higher photocatalyst concentrations (>0.2 g/L), the decrease observed in the PCO rate is possibly due to three nonmutually exclusive factors. One is the oversaturation of the solution with the photocatalyst, which results in photocatalyst agglomeration and decreases the surface area, leading to the absorption of most photons near the surface of the liquid hindering light penetration^55^ and hence lowering QE and reaction rates. This means that the As^III^ toward the bottom of the vessel is less likely to come into contact with the radical intermediates formed at the top of the vessel. Another possible factor is that a higher photocatalyst concentration results in a higher settling rate of the photocatalyst suspension. Finally, a last factor is that an increased concentration of the suspended photocatalyst in the solution results in more light scattering.^57^ All of these factors cause a slowdown in both the oxidation rate and QE (Figure 5D) at high Sn_1_Ti_1_O_2_ concentrations.

#### 3.2.5 Effect of the Initial As^III^ Concentration on the PCO Efficiency

The effect of the initial As^III^ concentration on the PCO process was studied for the concentration range 1 to 100 mg/L as presented in Figure S10. The results summary (Figure 5E) shows that the PCO rate constant decreases from (57 ± 3) × 10^–3^ min^–1^ (using As^III^ = 1 mg/L) to (1.9 ± 0.1) × 10^–3^ min^–1^ (using As^III^ = 100 mg/L) (Figure 5E). The lower PCO rate (*k*) observed at higher As^III^ concentration (while using fixed Sn_1_Ti_1_O_2_ concentration) is due to the higher As^III^ ions to unoccupied active sites ratio resulting in a high competition between As^III^ ions to bind to unoccupied sites. On the other hand, the results presented in Figure 5E show that there is a direct relationship between the quantum efficiency (QE%) and the initial concentration of As^III^, which increases from 0.22% at 1 mg/L to 1.08% at 100 mg/L. This is because at high concentrations there is a greater probability for As^III^ ions to interact with the charge carriers formed.^58^ This results in a greater kinetic competition for e_CB_^–^―h_VB_^+^ carriers to react with As^III^ species (either directly by h_VB_^+^, or indirectly by reactive oxygen species) and therefore results in a higher QE%. Overall, this shows that the photonic efficiency of the PCO of As^III^ is highly dependent on the concentration of As^III^ available for the reaction to occur.

### 3.3 Current Study and Literature

In this study, an assessment of the findings was carried out through a review of the literature focusing on TiO_2_-modified photocatalysts applied in the PCO of As^III^. The experimental setup for PCO exhibits a notable variety across the literature in operational parameters, such as irradiance power, light flux, As^III^ concentration, photocatalyst dosage, pH, and reaction duration. The differences in experimental setups cause challenges in directly comparing the efficiency of the photocatalysts themselves. Therefore, a detailed Table S3 incorporating these setup parameters was integrated to provide a comprehensive context for evaluating the performance of our novel Sn_1_Ti_1_O_2_ photocatalyst in relation to other photocatalytic systems. Nevertheless, the investigated Sn_1_Ti_1_O_2_ photocatalyst showcases significant promise when in contrast with the outcomes documented in previous studies.

### 3.4 Sn_1_Ti_1_O_2_ Recyclability

The recyclability of materials is a critical factor in evaluating their sustainability.^59^ The recyclability of the Sn_1_Ti_1_O_2_ heterojunction catalyst was investigated through three successive experiments. Initially, Sn_1_Ti_1_O_2_ was separated from the reaction mixture by using centrifugation. It was then washed several times with 0.1 M NaOH, followed by rinsing with deionized water to remove contaminants, ensuring that the wash water reached a neutral pH of 7.^60^ After washing, the catalyst was dried in a drying oven set to 70 °C to prevent structural damage. Finally, three successive photocatalytic reactions were conducted to assess the performance of the recovered Sn_1_Ti_1_O_2_ catalyst in the oxidation of As^III^. As shown in Figure S12, the photocatalytic efficiency slightly declined from 85.8% in the first cycle to 81.86% in the second cycle and further to 74.52% in the third cycle. These results confirm the practical applicability of Sn_1_Ti_1_O_2_ as a desirable photocatalyst that can be fully recovered and reused in a cost-effective manner.

## 4 Conclusion

The present work, for the first time, elucidated the PCO mechanism of As^III^ with Sn_1_Ti_1_O_2_ using transient absorption spectroscopy (TAS) over the microsecond time scale. We noted that (1) early time scale (pre-μs) electron–hole recombination was suppressed in Sn_1_Ti_1_O_2_, due to enhanced spatial separation of charge carriers; (2) enhanced hole scavenging effect in the presence of methanol, which pointed to the greater oxidative capability of the composite; (3) the As^III^ oxidation reaction in the presence of the colorimetric indicator molybdenum blue was studied in operando for the first time. A strong signal in the red region of the electromagnetic spectrum was observed in the Sn_1_Ti_1_O_2_ heterojunction from the start of our transient measurement (10 μs), which indicated that the oxidation of As^III^ to As^V^ and complexation of As^V^ with molybdenum blue occurs on the pre-microsecond time scale in this system. Focusing on Sn_1_Ti_1_O_2_, the main oxidant during the PCO of As^III^ to As^V^ was investigated using various scavengers. ^•^OH scavengers, such as isopropyl alcohol, rebamipide anhydrous, and DMSO, reduced PCO to 21, 30, and 23%, respectively. In contrast, O_2_^•—^ scavengers like SOD and *p*-benzoquinone showed lower suppression, reducing oxidation to 35 and 49%, indicating that ^•^OH is the primary reactive oxygen species (ROS) in the PCO of As^III^ with Sn_1_Ti_1_O_2_.

We further identified and examined the effects of experimental parameters on the PCO rate. A negative correlation was observed between the rate and salinity and As^III^ concentration, while a positive correlation was noted with irradiance power and pH. The QE positively correlated with pH and As^III^ concentration but negatively with salinity and irradiance power. The photocatalyst mass-to-solution ratio improved PCO rates and QE up to 0.2 g/L (the saturation limit) but had a negative impact beyond this mass. The Sn_1_Ti_1_O_2_ catalyst demonstrated practical recyclability, with the photocatalytic efficiency decreasing from 85.8 to 74.52% over three cycles, confirming its cost-effective reuse.

Overall, this detailed exploration of the Sn_1_Ti_1_O_2_ catalyst for the PCO of As^III^ to As^V^, including charge carrier dynamics, reaction kinetics, and identification of the main ROS, can aid in developing more efficient arsenic treatment facilities for water purification systems.

## Supporting Information Available

Additional experimental details include a summary of material characterization, *in situ* TAS figures for electron and hole scavenging experiments, baseline control experiments, and raw PCO results. A table compares the Sn_1_Ti_1_O_2_ photocatalyst’s performance against modified TiO_2_ systems reported in the literature for As^III^ oxidation. A figure summarizes the results of the reusability and recyclability of the Sn_1_Ti_1_O_2_ photocatalyst (PDF)
