# Supplementary material for: Role of the Sn-TiO2/Ti-SnO2 Heterojunction in Enhancing the Photocatalytic Oxidation of Arsenite (AsIII) through the Promotion of Charge Carrier Lifetime
Source: ACS Appl Mater Interfaces. 2024 Dec 4;16(50):69239–52. doi: 10.1021/acsami.4c14247 (PMC11660535; doi:10.1021/acsami.4c14247)
Supplement: Supplementary file 1 — am4c14247_si_001.pdf [file am4c14247_si_001.pdf]

Supporting Information

Title

The role of $Sn\text{-}TiO_2/Ti\text{-}SnO_2$ heterojunction in enhancing the photocatalytic oxidation of arsenite (As^{III}) through the promotion of charge carrier lifetime

Authors

Hany Fathy Heiba [a,b,c,d*], Jay C Bullen [a,b] , Andreas Kafizas [b,e], Camille Petit [f], Daqian Jiang [d], Dominik J Weiss [a*]

[a] Department of Earth Science & Engineering, Imperial College London, London SW7 2AZ, UK
[b] Department of Chemistry, Molecular Science Research Hub, Imperial College London, London W12 0BZ, UK
[c] National Institute of Oceanography and Fisheries, NIOF, Cairo 11516, Egypt
[d] Department of Civil, Construction, and Environmental Engineering, The University of Alabama, Tuscaloosa, AL 35487, USA
[e] Grantham Institute, Imperial College London, London SW7 2AZ, UK
[f] Barrer Centre, Department of Chemical Engineering, Imperial College London, London SW7 2AZ, UK

*Corresponding Author

Hany Fathy Heiba
[a] Department of Earth Science & Engineering, Imperial College London, London, SW7 2AZ, UK
[b] Department of Chemistry, Molecular Science Research Hub, Imperial College London, London, W12 0BZ, UK
[c] National Institute of Oceanography and Fisheries, NIOF, Cairo 11516, Egypt
[d] Department of Civil, Construction, and Environmental Engineering, The University of Alabama, Tuscaloosa, AL, 35487, USA
Email; hanyheiba@gmail.com; hfeiba@ua.edu; h.heiba17@imperial.ac.uk
ORCID: https://orcid.org/0000-0001-5253-5670

Dominik J Weiss
[a] Department of Earth Science & Engineering, Imperial College London, London, SW7 2AZ, UK
Email: d.weiss@imperial.ac.uk
ORCID: https://orcid.org/0000-0001-8074-9430

1 Chemicals and reagents

The list of chemical and suppliers are listed in table S1.

Table S1. List of chemicals used in this study.

Chemical	Purity/grade	Supplier company
Tin tetrachloride ($SnCl_4$)	98 %	Sigma Aldrich
Titanium(IV) tert-butoxide ($Ti[OC(CH_3)_3]_4$)	> 98 %	Sigma Aldrich
Ethanol (C_2H_5OH)	> 99.9 %	VWR
Arsenic trioxide (As_2O_3)	99.99% (trace metals basis)	Sigma Aldrich
Sodium Hydroxide (NaOH)	> 99 % (trace metals basis)	VWR
Hydrocholoric acid (HCl)	37 % trace metals basis	AnalaR
Nitric acid (HNO_3)	69 %	AnalaR
Ammonium molybdate ($(NH_4)_6Mo_7O_{24}$)	99.98% trace metals basis	Sigma Aldrich
Potassium antimony tartrate ($C_8H_4K_2O_{12}Sb_2$)	≥ 99 %	AnalaR
Sulphuric acid H_2SO_4	(95-99) %	VWR
Ascorbic acid ($C_6H_8O_6$)	≥ 99.0 % (ACS reagent)	Sigma Aldrich
Potassium permanganate ($KMnO_4$)	99 %	Sigma Aldrich
Sliver nitrate ($AgNO_3$)	≥ 99.0 % (ACS reagent)	Sigma Aldrich
Isopropanol (C_3H_8O)	≥ 99.9 % (HPLC grade)	VWR
Methanol (CH_3OH)	≥ 99.9 % (HPLC grade)	VWR
Rebamipide Anhydrous	≥ 98 % (HPLC)	Sigma Aldrich
Dimethyl Sulfoxide (DMSO)	> 99 % (HPLC grade)	VWR
Superoxide Dismutase (SOD)	Assay Kit	Sigma Aldrich
P-benzoquinone	≥98% (reagent grade)	Sigma Aldrich
Magnesium Chloride ($MgCl_2$)	99.99% (trace metals basis)	Sigma Aldrich
Sodium Sulfate (Na_2SO_4)	99.99% (trace metals basis)	Sigma Aldrich
Calcium Chloride ($CaCl_2$)	99.99% (trace metals basis)	Sigma Aldrich
Potassium Chloride (KCl)	99.99% (trace metals basis)	Sigma Aldrich
Potassium Bromide (KBr)	99.99% (trace metals basis)	Sigma Aldrich
Sodium Bicarbonate ($NaHCO_3$)	99.99% (trace metals basis)	Sigma Aldrich
Boric Acid (H_3BO_3)	99.99% (trace metals basis)	Sigma Aldrich
Strontium Chloride ($SrCl_2$)	99.99% (trace metals basis)	Sigma Aldrich
Sodium Fluoride (NaF)	99.99% (trace metals basis)	Sigma Aldrich

2 Summary of Materials Characterisation

An in-depth characterisation and metal oxide synthesis mechanism is included in our recently published study [1]. The key findings can be summarized as follows:

- XRD analysis confirmed the crystallinity of undoped anatase TiO_2, cassiterite SnO_2 and Sn-doped TiO_2 containing both cassiterite SnO_2 and anatase TiO_2 phases and was further confirmed by Raman spectroscopy.
- The addition of Sn into the TiO_2 lattice resulted in narrowing of the band gap with increasing Sn content. Band gap values decreased from 3.2 eV for pure TiO_2 to 2.9 eV for $Sn_1Ti_1O_2$, extending light absorption into the visible range. This was attributed to new intra-band states from Sn-induced unit cell expansion. The lowered band gap enhances visible-light photocatalytic activity by improving light harvesting efficiency.
- XPS confirmed Ti^{4+} and Sn^{4+} oxidation states. Ti 2p binding energies increased from 458.6 eV for TiO_2 to 459.0 eV for $Sn_1Ti_1O_2$, attributed to Fermi level shifts, as well as changes in C 1s and O 1s binding energies.
- LEIS showed that Sn predominates in the $Sn_1Ti_1O_2$ core while Ti floats on the surface.
- HR-TEM revealed pure anatase TiO_2 featured a 101 plane, whereas $Sn_1Ti_1O_2$ contained anatase TiO_2 101 planes amongst cassiterite SnO_2 110 particles. SnO_2 exclusively showed cassiterite 110 diffraction, confirming SnO_2 incorporation into TiO_2 with increased Sn content.

3 Results and discussion (SM)

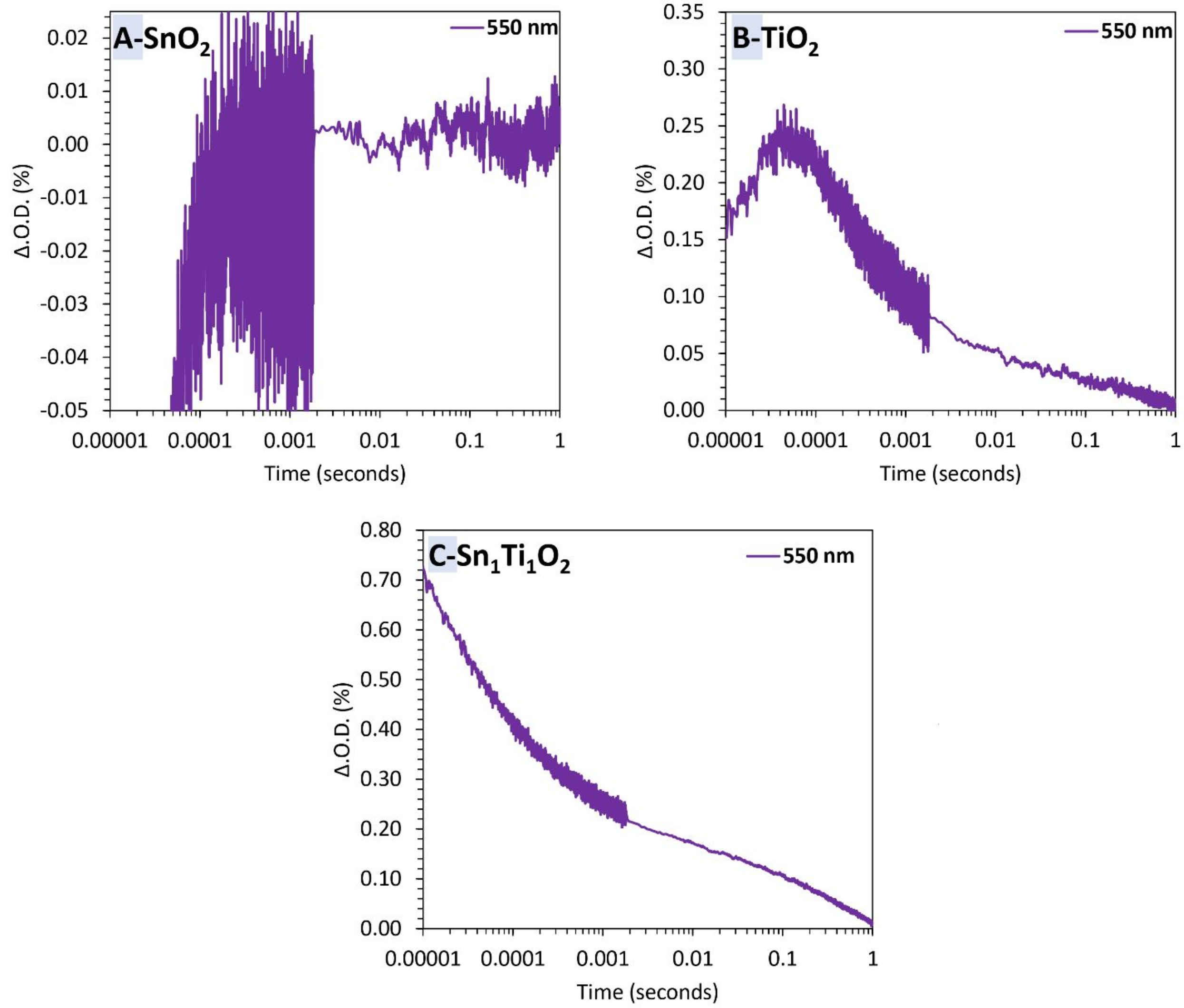

Figure S1. TAS of dry powder showing the changes in absorption vs time at 550 nm for SnO_2 (A), TiO_2 (B) and $Sn_1Ti_1O_2$ (C).

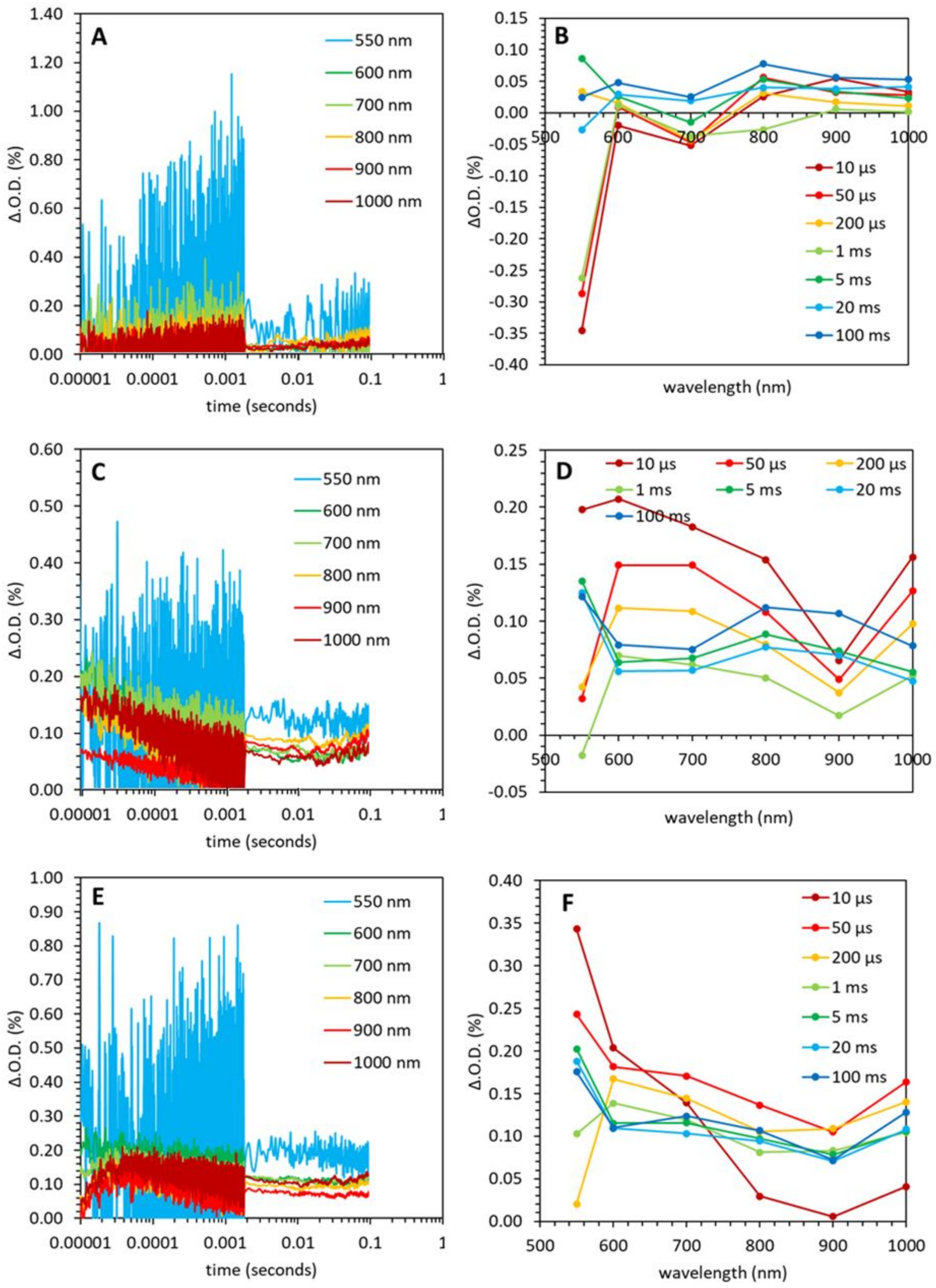

Figure S2. Transient absorption spectroscopy of powder suspensions (1g/L) in an aqueous $AgNO_3$ electron scavenger solution (2 mM $AgNO_3$; pH 7.3±0.1). Shown are changes in adsorption vs time for SnO_2 (A), TiO_2 (C) and $Sn_1Ti_1O_2$ (E) and changes in adsorption vs wavelength at select times for SnO_2 (B), TiO_2 (D) and $Sn_1Ti_1O_2$ (F). "The operating conditions; λ_{exc} = 355 nm, ~1.0 mJ cm^{-2} $pulse^{-1}$, 6 ns pulse width and laser repetition rate ~1 Hz".

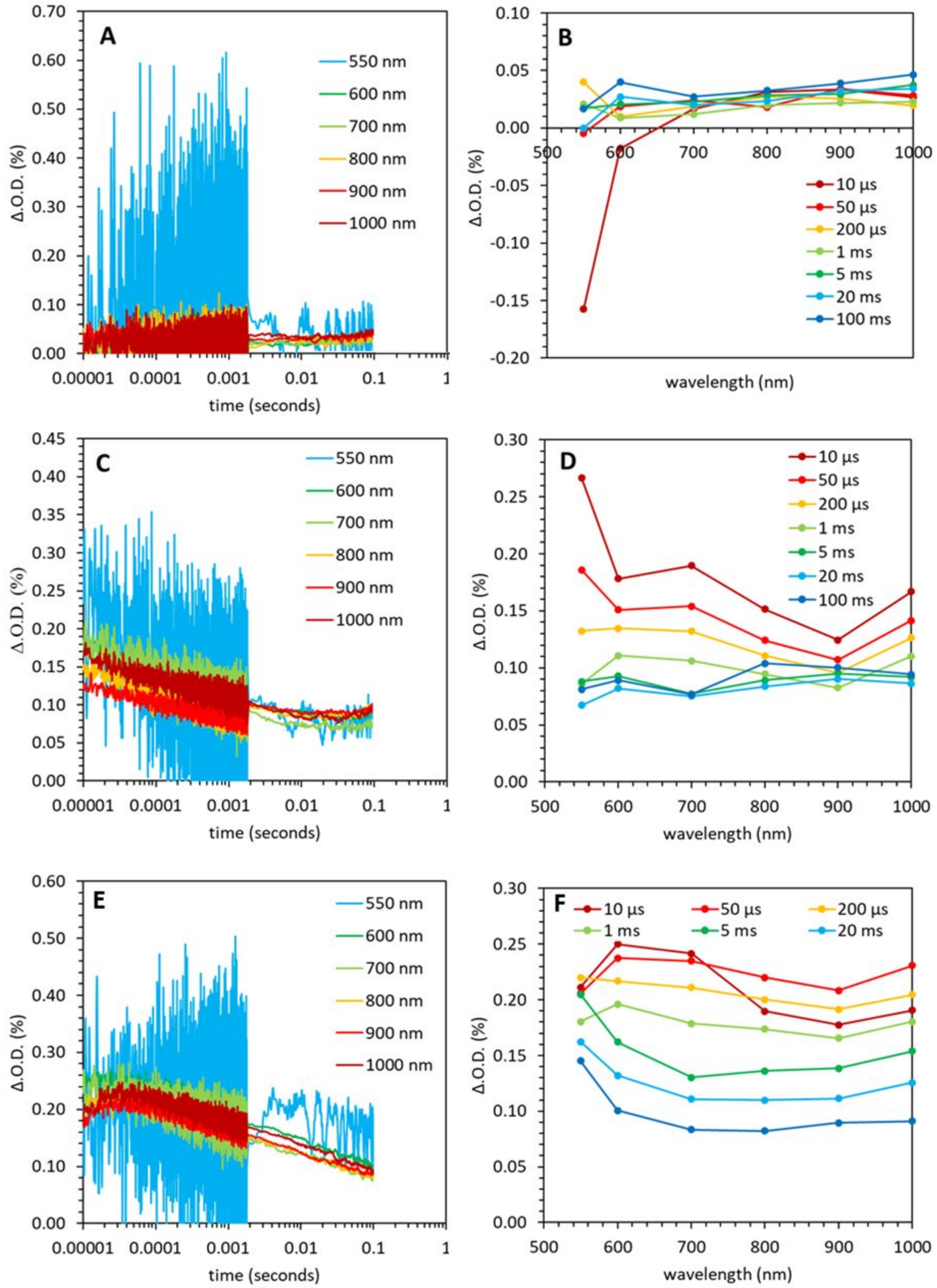

Figure S3. Transient absorption spectroscopy of powders suspensions (1g/L) in pure methanol hole scavenger solution. Shown are changes in adsorption vs time for SnO_2 (A), TiO_2 (C) and $Sn_1Ti_1O_2$ (E) and changes in adsorption vs wavelength at select times for SnO_2 (B), TiO_2 (D) and $Sn_1Ti_1O_2$ (F). "The operating conditions; λ_{exc} = 355 nm, ~1.0 mJ cm^{-2} $pulse^{-1}$, 6 ns pulse width and laser repetition rate ~1 Hz".

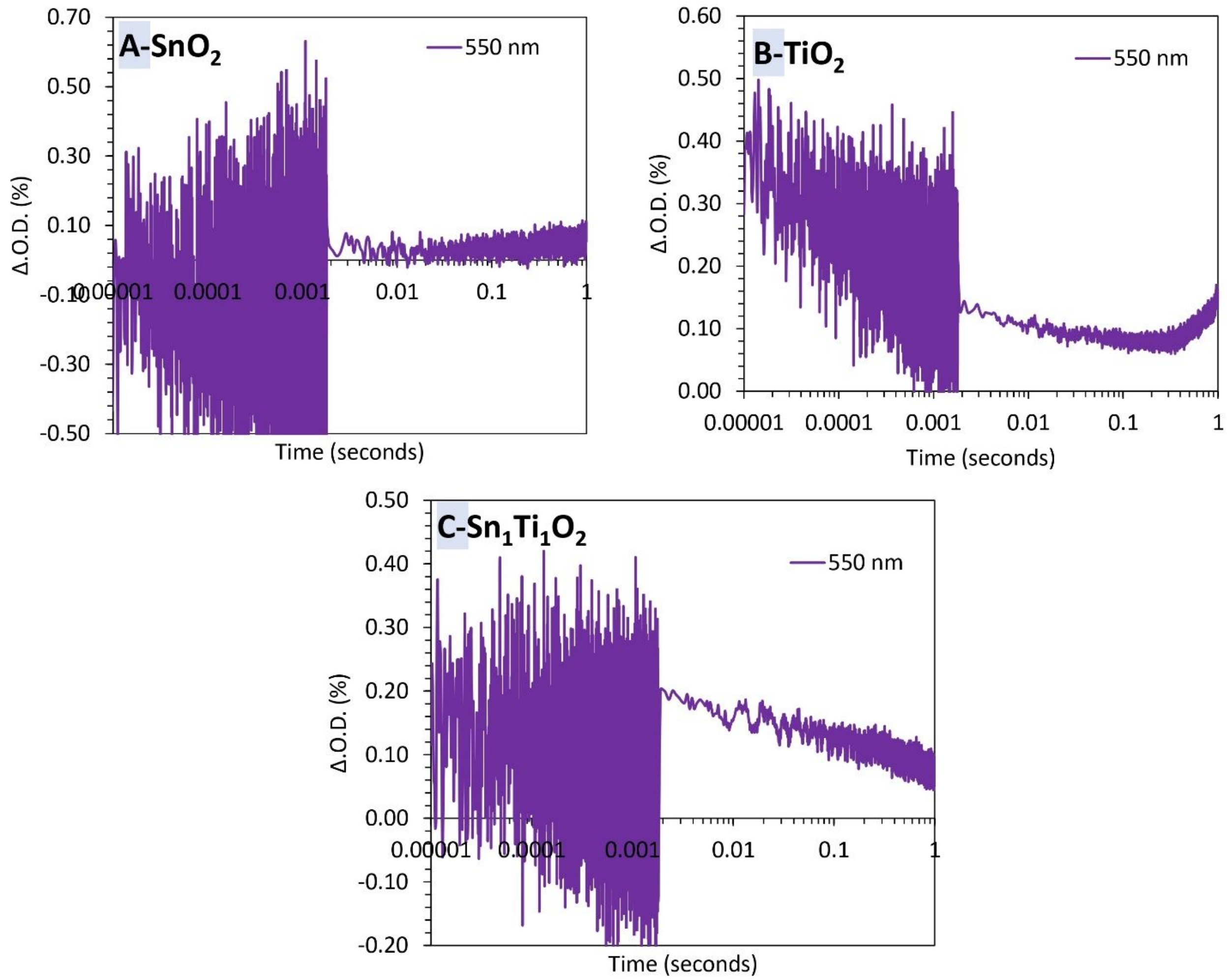

Figure S4 Transient absorption spectroscopy of powders suspensions (1g/L) in an aqueous solution containing 1 mg/L of As^{III} and molybdate complex that forms a blue coloured compound when bound to As^{V}. The changes in absorption vs time at 550 nm for SnO_2 (A), TiO_2 (B) and $Sn_1Ti_1O_2$ (C).

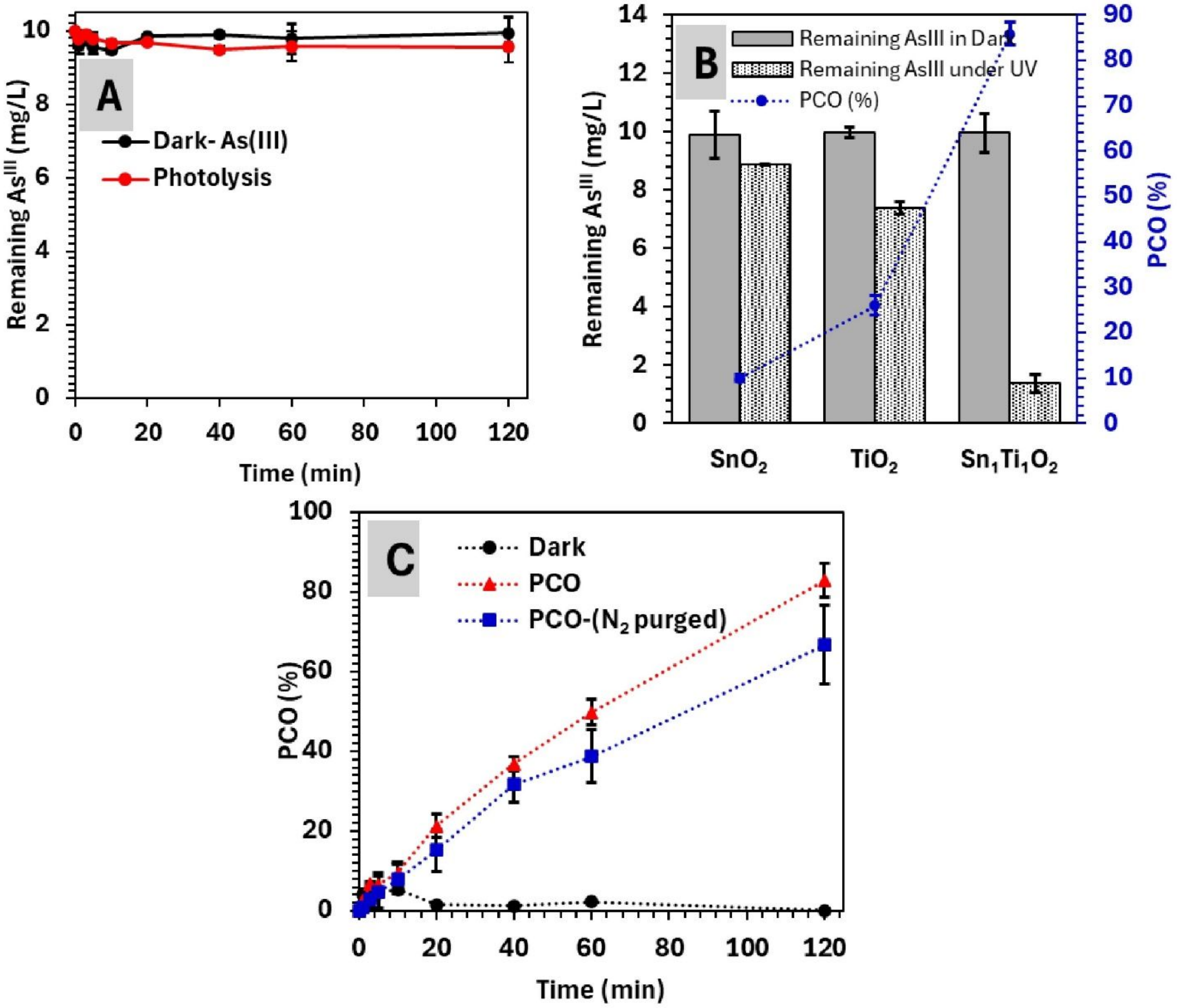

Figure S5. Baseline control experiments: (A) Adsorption in the dark using 10 mg/L As^{III} at pH 7.4 and direct UV photolysis using 7.40 mW/cm^2 irradiation, 10 mg/L As^{III} and pH 7.4. (B) PCO using SnO_2, TiO_2, and $Sn_1Ti_1O_2$ showing the decrease in As^{III} in the dark and under PCO, in addition to PCO % using 10 mg/L As^{III}, 0.1g/L photocatalyst, pH 7.4, and 7.40 mW/cm^2 irradiation. (C) Effect of dissolved O_2 on the PCO using a N_2-purged solution prior to PCO for 20 minutes (20 mL N_2/s), 10 mg/L As^{III}, 0.1g/L $Sn_1Ti_1O_2$, pH 7.4, and 7.40 mW/cm^2 irradiation.

Table S2. Effect of scavengers on the PCO of As^{III}

	DW	ECB	HVB	OH· scavenger			$O^{\cdot}_2$ scavenger	
	non	$AgNO_3$	Ascorbic Acid	Isopro panol	Rebamipide Anhydrous	DMSO	SOD	p-benzoquinone
Oxidation % (after 2 hrs)	62.43	85.25	2.33	21.01	30.25	23.10	35.25	48.93
Change in Oxidation %	n/a	36.55	-96.27	-66.35	-51.55	-63.00	-43.53	-21.62
k_1	0.008	0.015	0.002	0.004	0.003	0.003	0.004	0.006
Standard Error (SE)	1.9 x 10^{-4}	8.9 x 10^{-4}	1.4 x 10^{-4}	3.6 x 10^{-4}	2.6 x 10^{-4}	2.9 x 10^{-4}	1.7 x 10^{-4}	4.3 x 10^{-4}
QE*(%)	0.64	0.84	0.18	0.42	0.33	0.32	0.37	0.56
RSS**	0.00	0.07	0.00	0.00	0.01	0.00	0.00	0.02
Pearson's r	-1.00	-0.99	-0.98	-0.97	-0.98	-0.97	-0.99	-0.98
R^2	1.00	0.97	0.95	0.94	0.95	0.94	0.98	0.96

* Represents the quantum efficiency, ** Relative Standard Deviation

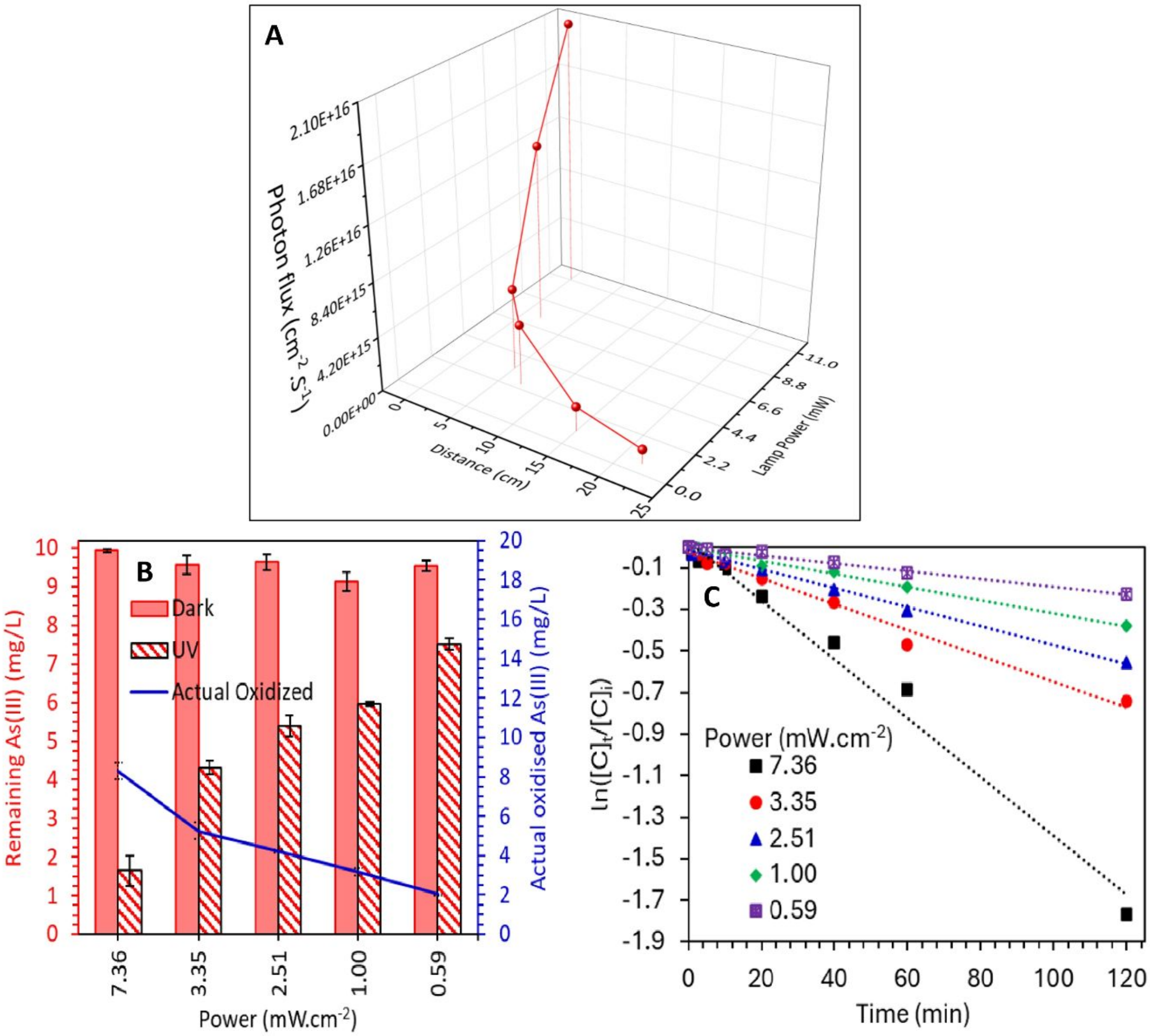

Figure S6. Effect of distance between the irradiation source and the solution surface on the lamp power and the photon flux yield (A). Effect of irradiation power on the PCO efficiency of As^{III} (B & C). Remaining As^{III} under UV and in dark and the actual oxidised As^{III} after 2 hrs (B), First kinetic order plot at different irradiation power (C).

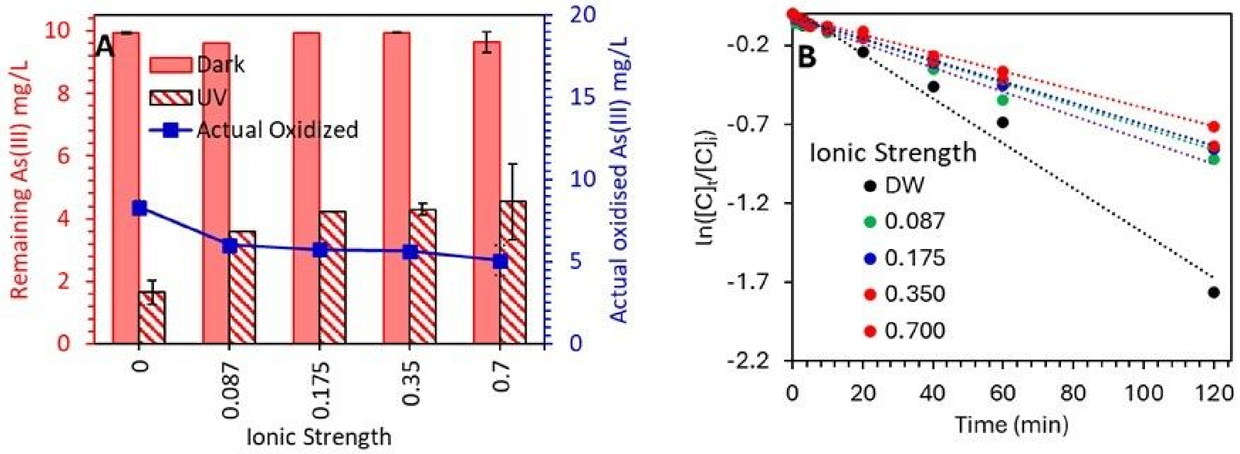

Figure *S7*. *Effect of salinity on the PCO of* As^{III}. *Remaining* As^{III} *in solution under UV and in dark and the actual oxidised* As^{III} *portion after 2 hrs (A), First order kinetic plots for different ionic strengths (B).*

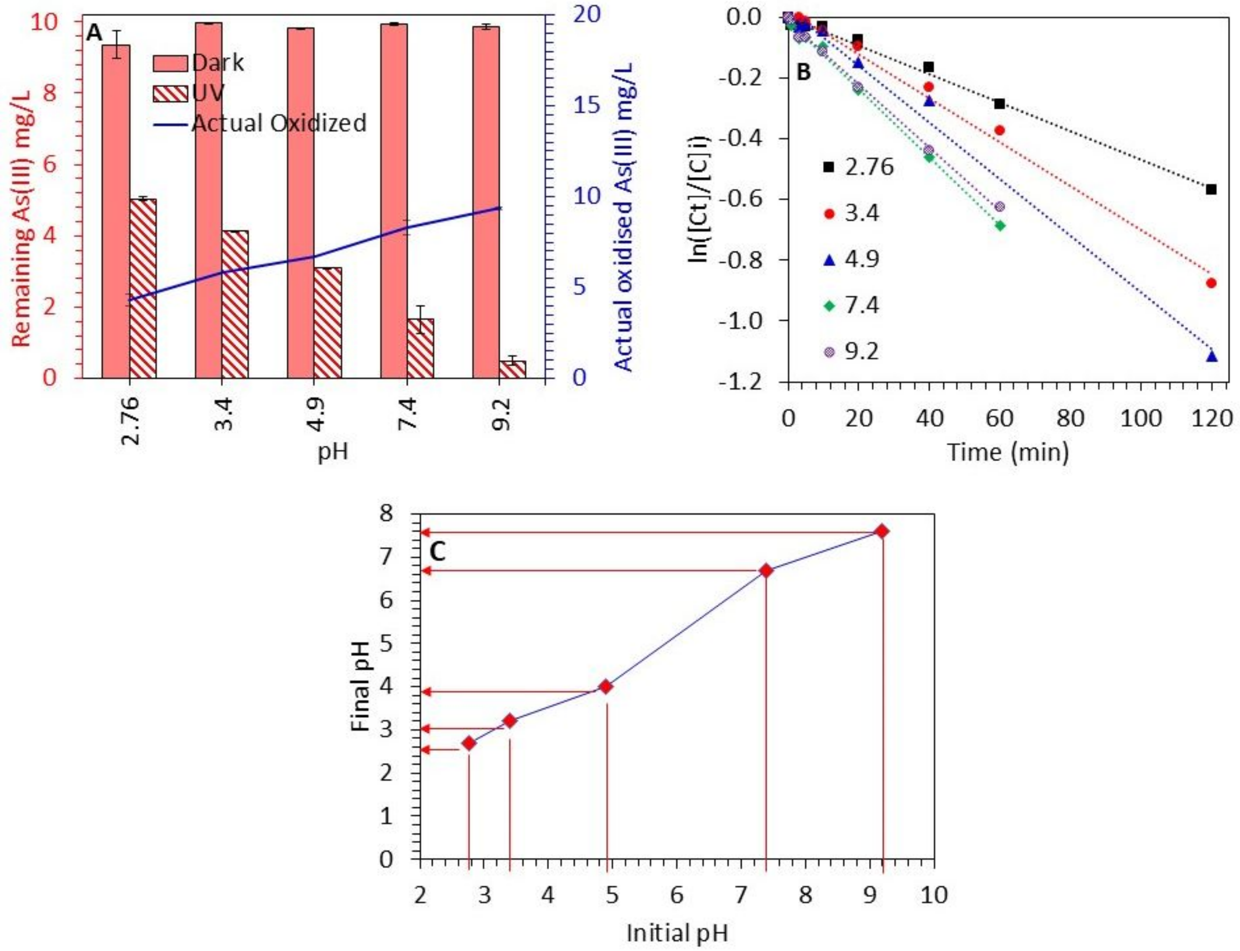

Figure S8. Effect of pH on the PCO of As^{III}. Remaining As^{III} under UV and in dark and the actual oxidised As^{III} after 2 hrs (A), First order kinetic plots at different pH (B), Change in pH after conducting the PCO experiment (C).

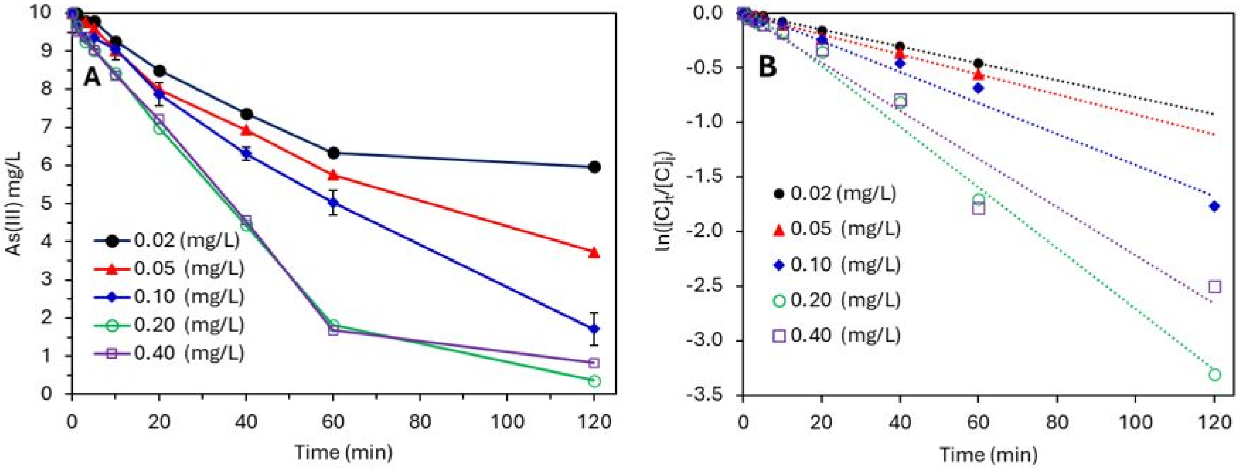

Figure S9. Effect of mass to solution ratio on the PCO of As^{III}. As^{III} in solution after correcting for adsorption (A), and the first order kinetic plot (B). The experiments were performed using 100 ml of 10 mg/L As^{III}, pH 7.4 adjusted with using 1 M HCl and 1 M NaOH, catalyst dose (0.02, 0.05, 0.1, 0.2,0.4 mg/L). Error bars represent the ± SD.

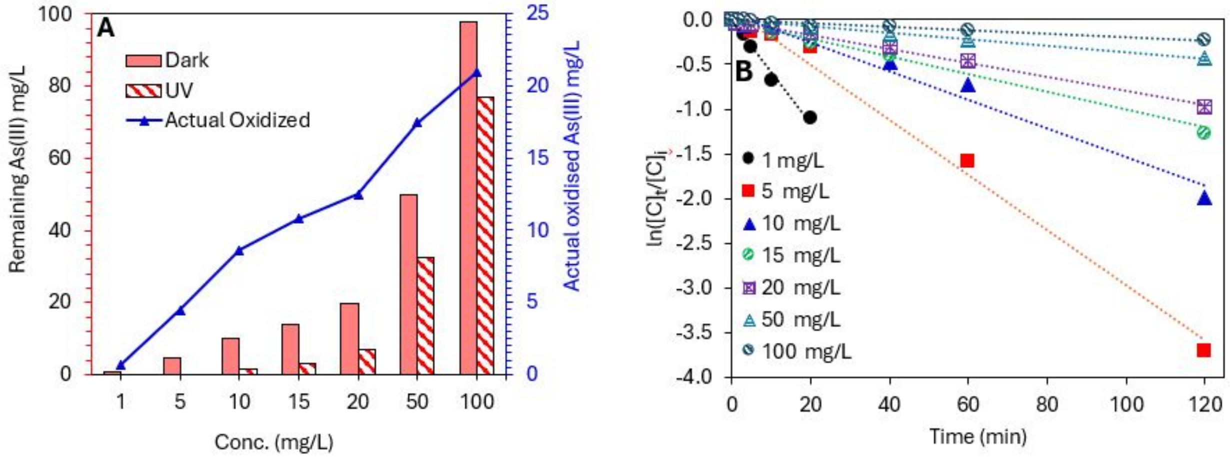

Figure S10. Effect of As^{III} concentration on the PCO of arsenite As^{III}. Remaining As^{III} in solution under UV and in dark and the actual oxidised As^{III} after 2 hrs (A), First order kinetic plots for different As^{III} concentrations (B).

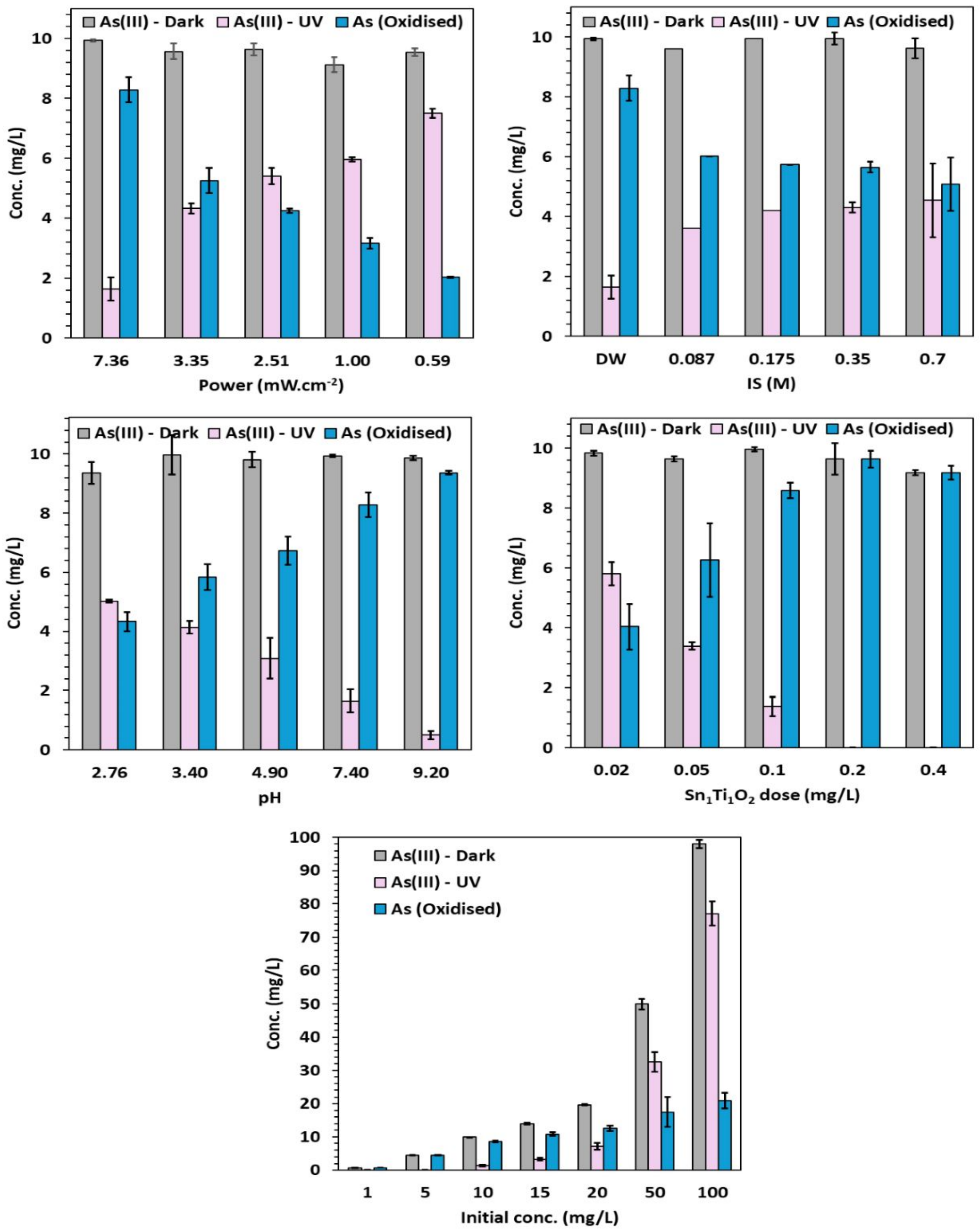

Figure S11. Summary of the remaining [As^{III}] after 120 min of dark and UV irradiation, and the absolute oxidized [As^{III}], utilizing the $Sn_1Ti_1O_2$ photocatalyst system under varied operational conditions: A) Irradiance Power (10 mg/L [As^{III}], pH 7.4, 0.1g/L $Sn_1Ti_1O_2$); B) Ionic Strength (10 mg/L [As^{III}], pH 7.4, 0.1g/L $Sn_1Ti_1O_2$, irradiation 7.40 mW/cm^2); C) pH (10 mg/L [As^{III}], 0.1g/L $Sn_1Ti_1O_2$, irradiation 7.40 mW/cm^2); D) $Sn_1Ti_1O_2$ dosage (10 mg/L[As^{III}], pH 7.4, irradiation 7.40 mW/cm^2); E) Initial [As^{III}] concentration (pH 7.4, 0.1g/L $Sn_1Ti_1O_2$, irradiation 7.40 mW/cm^2). The error bars indicate the standard deviation (±SD).

Table S3. A comparison of the $Sn_1Ti_1O_2$ photocatalyst's performance against modified TiO_2 systems reported for As^{III} oxidation in the literature.

Catalyst	Experimental Conditions					Oxidation		Ref
	Photocatalyst mass [g/L]	pH	Irradiation Source	Time	Initial $[AS^{III}]$ mg/L	Oxidized $[As^{III}]$ mg/L	%	
$NiFe_2O_4/TiO_2$	1	*NM	Hg vapor lamp (32.62 mW cm^{-2}, 250 W)	80 min	0.2	0.195	97.5	Borges et al. [60]
TiO_2/Activated Carbon Fiber	3	7	UV Hg lamp (125 W, 365 nm)	120 min	3.0	0.5	83.33	Xiao et al. [61]
PAN@Ag–Ag_2O/TiO_2	0.12	5	Xe lamp (500 W)	120 min	5.0	4.61	92.1	Ren et al. [62]
TiO_2	1	*NM	Low-pressure Hg lamps (1.5–5 × 10^{16} (photon/s)/cm^3) blazed at 350 nm	15 min	1.0	> 0.904	>90	Xu et al. [41]
Pt/TiO_2	*NM	7	High-pressure Hg lamp (175-W)	280 min	3.2	2.59	82	Qin et al. [63]
TiO_2 fibres calcined at 700 °C<br>TiO_2 anatase<br>N-doped P25 TiO_2<br>N-doped TiO_2 calcined at 500 °C	0.5	7	Xe lamp, (15 µW cm^{-2}, 500 W)	90 min	10	8<br>3.5<br>7<br>7	80<br>35<br>70<br>~70	Zhang et al. [64]
N-doped TiO_2	0.05	2	Xenon arc lamp; λ > 309 nm (light flux 365 nm, 1.56 mW cm^{-2}, 450 W)	4 hr	3.0	2.64	~ 88	Wang et al. [24]
γ-Fe_2O_3@PANI@TiO_2	1	5	Xe lamp (500 W)	300 min	5.0	3.75	75	Wang et al. [65]
meso-TiO_2/Fe_2O_3	0.1	7.3	UV lamp (λ=368 nm, 18 mW)	120 min	1.0	0.98	≥ 98	Bullen et al. [5]
TiO_2	0.1	7.4	UV lamp (λ=368 nm, 7.4 mW)	120 min	10.0	2.6	26	Current Study
SnO_2	0.1	7.4	UV lamp (λ=368 nm, 7.4 mW)	120 min	10.0	1.3	13	Current Study
$Sn_1Ti_1O_2$	0.1	7.4	UV lamp (λ=368 nm, 7.4 mW)	120 min	5.0<br>10.0<br>15.0<br>20.0<br>50.0<br>100.0	4.486<br>8.63<br>10.78<br>12.49<br>17.43<br>20.93	89.7<br>86.3<br>71.9<br>62.4<br>34.9<br>20.9	Current Study

*NM= Not Mentioned

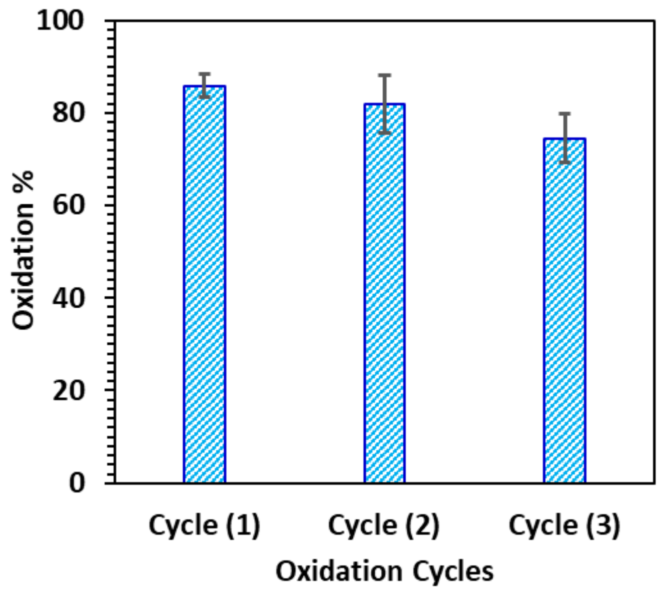

Figure S12. The reusability and recyclability of the $Sn_1Ti_1O_2$ photocatalyst over three consecutive PCO cycles.
